# A Novel CAR Expressing NK Cell Targeting CD25 With the Prospect of Overcoming Immune Escape Mechanism in Cancers

**DOI:** 10.3389/fonc.2021.649710

**Published:** 2021-05-14

**Authors:** Moein Dehbashi, Zohreh Hojati, Majid Motovali-bashi, Mohamad Reza Ganjalikhany, William C. Cho, Akihiro Shimosaka, Parnian Navabi, Mazdak Ganjalikhani-Hakemi

**Affiliations:** ^1^ Division of Genetics, Department of Cell and Molecular Biology and Microbiology, Faculty of Biological Science and Technology, University of Isfahan, Isfahan, Iran; ^2^ Division of Biochemistry, Department of Cell and Molecular Biology and Microbiology, Faculty of Biological Science and Technology, University of Isfahan, Isfahan, Iran; ^3^ Department of Clinical Oncology, Queen Elizabeth Hospital, Hong Kong, China; ^4^ Institute of Hematology, Peking Union Medical College, Beijing, China; ^5^ Department of Immunology, Faculty of Medicine, Isfahan University of Medical Sciences, Isfahan, Iran; ^6^ Acquired Immunodeficiency Research Center, Isfahan University of Medical Sciences, Isfahan, Iran

**Keywords:** cancer, chimeric antigen receptor, regulatory T cells, CD25, NK-92

## Abstract

For many years, high-affinity subunit of IL-2 receptor (CD25) has been considered as a promising therapeutic target for different pathologic conditions like allograft rejection, autoimmunity, and cancers. Although CD25 is transiently expressed by newly-activated T cells, it is the hallmark of regulatory T (Treg) cells which are the most important immunosuppressive elements in tumor microenvironment. Thus, Tregs can be considered as a potential target for chimeric antigen receptor (CAR)-based therapeutic approaches. On the other hand, due to some profound adverse effects pertaining to the use of CAR T cells, CAR NK cells have caught researchers’ attention as a safer choice. Based on these, the aim of this study was to design and develop a CAR NK cell against CD25 as the most prominent biomarker of Tregs with the prospect of overcoming immune escape mechanism in solid and liquid cancers. In the current study, an anti-CD25 CAR was designed and evaluated by comprehensive *in silico* analyses. Then, using lentiviral transduction system, NK-92 cell line was engineered to express this anti-CD25 CAR construct. *In vitro* functional analyses of anti-CD25 CAR for its reactivity against CD25 antigen as well as for cytotoxicity and cytokine production assays against CD25 bearing Jurkat cell line were done. *In silico* analyses demonstrated that the anti-CD25 CAR transcript and scFv protein structures were stable and had proper interaction with the target. Also, *in vitro* analyses showed that the anti-CD25 CAR-engineered NK-92 cells were able to specifically detect and lyse target cells with an appropriate cytokine production and cytotoxic activity. To conclude, the results showed that this novel CAR NK cell is functional and warrant further investigations.

## Introduction

The cytokine interleukin (IL)-2, also called “T-cell growth factor,” is a potent stimulator for *in vitro* expansion of T cells. Activation of Tcells *via* T-cell receptor (TCR) lead to expression of high levels of a high affinity IL-2 receptor (IL-2R) (1). This receptor is composed of three noncovalently bound subunits including an α chain (also called CD25), a β and a α chain. CD25 is transiently expressed on almost all activated T cells but not on resting ones. The interaction of IL-2 with CD25 is necessary for the clonal expansion and viability of activated T cells (2, 3). However, persistent high expression of CD25, as well as expression of CD4, and intracellular expression of FoxP3 is the hallmark of Tregs (4). The role of IL-2 in the development and homeostasis of Tregs is critical and hence, most of these cells express high levels of CD25 on their surface (5). Tregs inhibit the expansion of effector T cells *in vivo* and hence, suppress the autoimmune reactions as well as tumor immune surveillance, and antitumor immune responses. The uppermost suppression levels are likely mediated by Tregs expressing high levels of CD25. Particularly, Tregs can markedly inhibit CD8^+^ T cells activation and cause immune dysfunction in cancer patients. Thus, Tregs are the major obstacle for the successful development of cancer immunotherapy (6). Based on these, CD25 has been used as a promising therapeutic target for the treatment of wide-range of pathologic conditions such as organ transplantation, graft versus host disease (GVHD), autoimmunity, and cancers.

Daclizumab is a humanized monoclonal antibody (mAb) of IgG1 isotype specific for the alpha subunit (CD25) of the IL-2 receptor that effectively blocks its interaction with IL-2 (1, 3). In the context of organ transplantation, allograft rejection is associated with an enhanced serum-soluble CD25 level that is related to major histocompatibility complex (MHC) mismatch recognition by T cells and their activation. In several pre-clinical studies as well as in clinical trials involving a large group of recipients, daclizumab, as an efficacious CD25 blocker, has reduced the rejection episodes in patients receiving cardiac, liver, kidney, and pancreatic islet transplants (2, 7–10).

Most of studies on blocking IL-2/CD25 interaction in autoimmune diseases have been done on multiple sclerosis (MS). It has been shown that, daclizumab therapy can provide multifactorial immunomodulatory impacts with consequential suppression of inflammatory responses in MS and reduced disease activity to a greater extent than interferon beta alone (1, 11).

Harnessing the immune responses as a therapeutic approach for cancer is the main purpose of tumor immunotherapy. Tumors recruit cancer immune escape mechanisms which play an important role to abrogate a successful treatment (12, 13). It is noteworthy that the immune cells including CD4^+^CD25^+^FOXP3^+^Treg cells, myeloid-derived suppressor cells (MDSCs) and tumor-associated macrophages (TAMs) type 2 are engaged in this mechanism by infiltrating into tumors and hindering antitumor immune responses of tumor antigen-specified CD8^+^ T cells and CD4^+^ T cells (14). Amongst, CD4^+^CD25^+^ Tregs cells within tumor microenvironment (TME) called tumor-infiltrating Treg cells (TI-Tregs) have the critical role in cancer immune escape (15). Therefore, a vast majority of research and clinical trials has been done on the evaluation of the therapeutic potential of daclizumab or other CD25 blocking agents in cancers. It has been shown that targeting CD4^+^CD25^+^ Tregs cells can improve the chance of successful cancer treatment and tumor rejection responses (16). It has been shown that daclizumab can markedly deplete CD25^+^ FOXP3^+^ CD4^+^ T cells from the peripheral blood for 3 to 4 weeks with the recovery of Treg phenotype and function by 8 weeks (17, 18). Depletion of Treg cells *in vivo* increases anti-tumor immunity in numerous animal models (19–21). Applying mAbs like daclizumab, is undergoing clinical trials (6) and a number of Treg-depleting strategies have been evaluated in clinical trials. In an improved strategy, injection of animal models with anti-CD25 antibody depletes animals of CD25^+^ Tregs and increases the response to cancer vaccination (22, 23). Some clinical studies have examined anti-CD25 recombinant immunotoxins, like Denileukin diftitox, as Treg depleting agents. A marked depletion of the numbers of CD25^+^ FOXP3^+^ CD4^+^ T cells, both in blood and tumors of patients with melanoma, renal carcinoma, and ovarian cancer, was observed. However, the effect was highly transient and rarely persist for more than three weeks as the half-life of one immunotoxin is only 2 h (4, 24–29).

Regardless of their beneficial therapeutic effects, using mAbs can provoke a wide variety of systemic and local adverse events from infusion reactions (IRs), headache, mild gastrointestinal symptoms, transient urticaria, rash and itching, to increased risk of fatal infections, anaphylaxis, immune thrombocytopenia, neutopenia, hemolytic anemia, vasculitis, serum sickness, cytokine release syndrome (CRS), tumor lysis syndrome (TLS), autoimmune diseases, Stevens-Johnson syndrome (SJS), toxic epidermal necrolysis (TEN), heart, pulmonary, hepatic, kidney, embryo-fetal, and neurological toxicities (1, 10, 30). In addition, regarding the fact that mAbs require the engagement of components of the host immune system to spark complement dependent cytotoxicity (CDC), and antibody-dependent cellular phagocytosis/cytotoxicity (ADCP/ADCC) (30, 31) and as the host immune system is usually compromised in cancer patients, there is a need for other strategies which are not rely on the host immune system and besides, having less adverse reactions.

Nowadays, beyond mAbs and antibody-drug conjugates, cancer immunotherapy-based CAR has shown promising results in approved treatments and clinical trials. A great bulk of works has been done using CAR expressing T cells in cancer treatment research. However, the main obstacle of using anti-cancer CAR T cells is their dangerous adverse effects that encourages scientists to apply NK cells as a safer alternative. One drawback of CAR T cells as opposed to CAR NK cells is that CAR T cells can cause cytokine-related adverse events (cytokine storm) in the clinical application while NK cells could be safer effector cells due to the lack of clonal expansion. Moreover, production of autologous CAR T cells is an expensive and time-consuming method, while CAR NK cells could be applied in the setting of allogeneic transplantation, without inducing GVHD (32, 33). In recent years, progress in the biology field of NK cells and better understanding of NK cells function, has resulted in developing NK cells as a promising tool for cancer immunotherapy (34). Hence, this work was focused on making a CAR NK cell by genetically manipulating NK-92 cell line for expressing a third-generation CAR. Here, theaim was to design as well as *in silico* and *in vitro* evaluation of the novel anti-CD25 CAR-engineered NK-92 cell with the prospect of overcoming immune escape mechanism in solid and liquid cancers.

## Methods

### Generation of the Anti-CD25 CAR Lentiviral Construct

**Figure 1:**
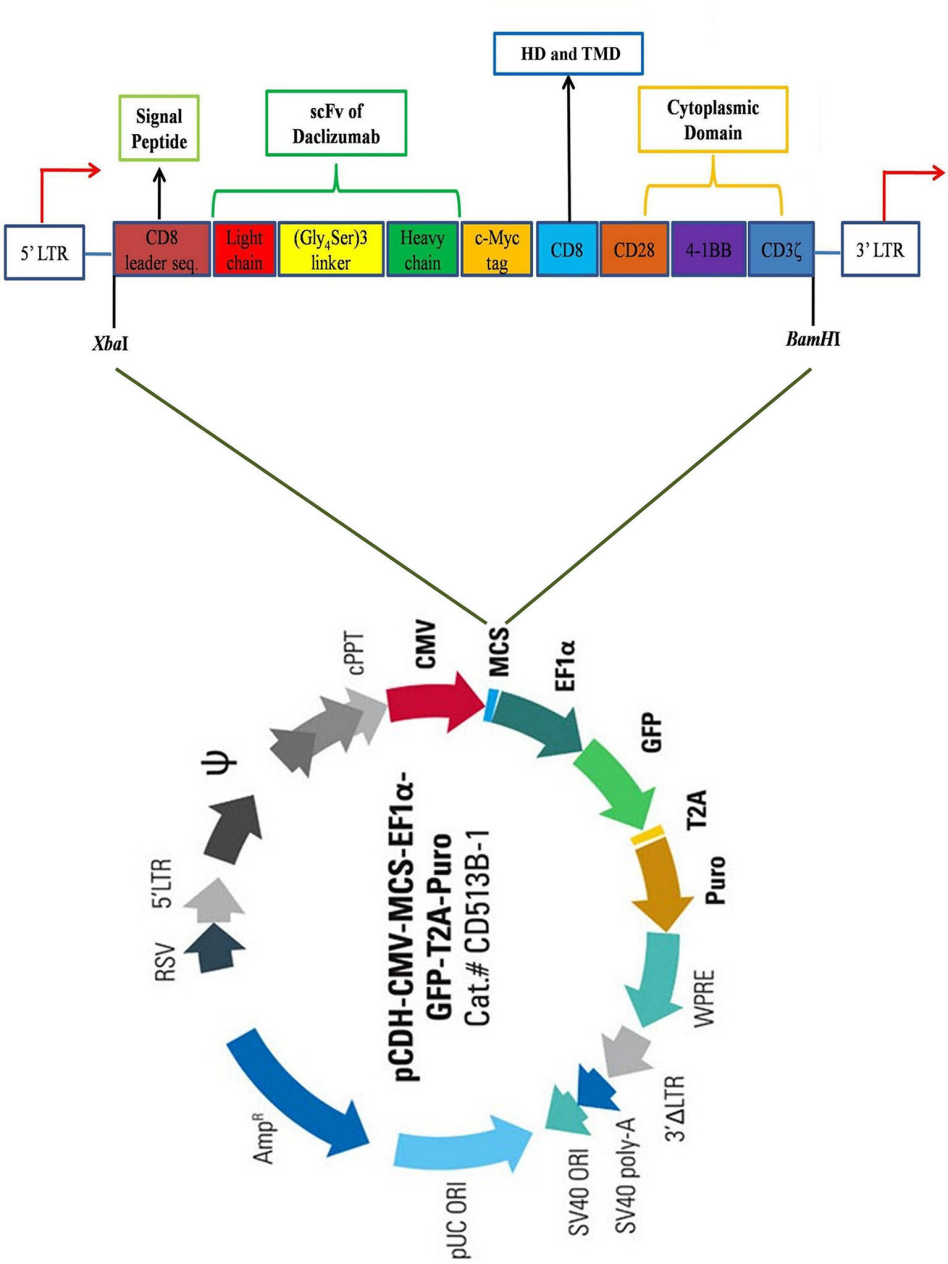
Schematic view of the CAR construct. The construct contains a CD8 leader sequence, a scFv of daclizumab containing (Gly_4_Ser)_3_ linker, a c-Myc tag, a CD8 hinge region, as well as CD28, 4-1BB, and CD3ζ signaling domains, respectively from 5′ to 3′ end. The construct was inserted in the multiple cloning site (MCS) of pCDH-513B transfer vector (System Bioscience, USA), between *Xba*I and *Bam*HI restriction sites. HD, Hing domain; TMD, Transmembrane domain.

Firstly, the protein sequences of the hinge and transmembrane part of CD8, as well as cytosolic signaling domains of CD28, 4-1BB, and CD3ζ retrieved from UniProt (www.uniprot.org) and converted to the corresponding DNA sequence by using EMBOSS Backtranseq (www.ebi.ac.uk/Tools/st/emboss_backtranseq). The daclizumab scFv amino acid sequence from PDB ID: 3NFP containing the linker of (Gly_4_Ser)_3_ was applied as the antigen binding domain of the construct. A c-Myc TAG sequence was placed immediately after scFv heavy chain (VH) encoding sequence. In addition, the signal peptide (SP) sequence of CD8 was used as the SP of the CAR to conduct it to express as a membrane bound protein. To enhance the expression of the construct gene, the Kozak sequence, GCCACC, was added at the beginning of construct (5′ end) just before CD8 SP sequence. The restriction maps were studied in NEBcutter2 (http://nc2.neb.com/NEBcutter2), then, *Xba*I and *BamH*I were selected as restriction enzymes for 5′ and 3′ ends, respectively. Finally, the construct sequence was ordered to be made in PCDH-513B transfer vector (System Bioscience, USA) by the General Biosystem company (USA) (**Figure 1**).

### Computational Analysis of the Designed Anti-CD25 CAR

The daclizumab scFv amino acid sequence from PDB ID: 3NFP containing the linker of (Gly_4_Ser)3 was submitted to Robetta web server (http://robetta.bakerlab.org) to obtain the predicted 3D models. The SWISS-MODEL web server (https://swissmodel.expasy.org) was used to fix the structural breaks of CD25 from PDB ID: 1Z92. The PRISM web server (http://cosbi.ku.edu.tr/prism) was utilized to predict the interaction models using the available and relative structures between the scFv and CD25 structures. Also, Swiss PDB Viewer v4.1 software (https://spdbv.vital-it.ch) was applied for further structural investigations. Using VADAR version 1.8 (http://vadar.wishartlab.com), RAMPAGE (http://mordred.bioc.cam.ac.uk/~rapper/rampage.php), Ramachandran plot (https://swift.cmbi.umcn.nl/servers/html/ramaplot.html) and Web-based PDB structural analysis service (http://tomcat.cs.rhul.ac.uk/home/mxba001), the φ, ψ and ω torsional angles and accessible surface area (ASA) of the scFv were measured. Furthermore, in order to evaluate the contact and distance maps, NAPS (http://bioinf.iiit.ac.in/NAPS) was utilized. Then, the molecular dynamics (MD) simulation has been performed by using Amber V.14 and ff14SB as force field (35). The MD simulation of the complex structure (scFv-CD25) has been performed for 170 ns. The complex structure was solvated in truncated octahedron box with TIP3P water model in a 10 Å hydration layer. Minimization of the system was performed for 5,000 steps. The system was heated from 0 to 300 K for 300 ps, using Langevin thermostat in NVT ensemble. The equilibration was performed in two steps, for 150 and then 500 ps in NVT and NPT ensemble respectively. Finally, production MDs was done in 170 ns in periodic boundary condition and Particle Mesh Ewald (PME) method was used for calculation of non-bonded interactions with 10 Å cutoff. In order to analyze the MD simulation, CPPTRAJ program from AMBER Tools V.14 was used. The root mean square deviation (RMSD), fluctuation (RMSF) and H-bonds analysis were carried out for the complex structure.

### Glycosylation Prediction

To predict the glycosylation chains for amino acid residues, NetNGlyc 1.0 Server (http://www.cbs.dtu.dk/services/NetNGlyc) and NetOGlyc 4.0 Server (http://www.cbs.dtu.dk/services/NetOGlyc) were applied. Both these web servers are related to the comprehensive ExPASy web server (https://www.expasy.org).

### Cell Culture

The Lenti-X 293T cell line (Clontech, USA) was cultured in Dulbecco’s Modified Eagle’s Medium (DMEM) supplemented with 10% fetal bovine serum (FBS) (Biowest, France) and 1% penicillin-streptomycin (PenStrep) solution. The Jurkat cell line (E6-1) (a kind gift of Dr. Yousof Gheisari) was maintained in RPMI 1640 supplemented with 10% FBS and 1% PenStrep. The NK-92 cell line which wasa gift of Prof. Dr. Matthias Schneider cultured in Alpha Minimum Essential Medium (Alpha MEM) (Sigma-Aldrich, USA) without ribonucleosides and deoxyribonucleosides but with 2 mM L-glutamine and 1.5 g/L sodium bicarbonate. To make the complete growth medium, the following components were added to the base medium: 0.2 mM inositol, 0.1 mM 2-mercaptoethanol, 0.02 mM folic acid, 100-200 U/mL recombinant IL-2, 1% PenStrep and adjusted to a final concentration of 12.5% horse serum (Sigma-Aldrich, USA) and 12.5% FBS. The cultured cell lines were kept at 37°C in a humidified incubator with 5% CO_2_.

### Production of Lentiviruses Containing Anti-CD25 CAR

The anti-CD25 CAR (GenBank accession number of MT780308) inserted between *Xba*I at 5′ and *Bam*HI at 3′ sites in the HIV-1-based, VSV-G pseudo typed lentiviral vector, pCDH-513B-1 (System Bioscience, USA) containing CMV-MCS-EF1α-copGFP-T2A-Puro to release the 1,551-bp fragment. In order to produce lentiviruses, Lenti-X 293T, ps-PAX2.2, and pMD2.G as packaging cell line, packaging vector, and envelope vector were used, respectively. Briefly, Lenti-X 293T cell line was cultured in a T75 culture flask at a density of 6 × 10^6^ in 8 mL DMEM medium containing 10% FBS and 100 U/mL penicillin/streptomycin. In order to produce the lentiviruses, co-transfection was done by adding 21 μg packaging vector of ps-PAX2.2 (Addgene, USA), 10.5 μg envelope vector of pMD2.G (Addgene, USA) and 21 μg transfer vector (pCDH-anti-CD25 CAR) into Lenti-X 293T cells by the calcium phosphate precipitation method and after 16 h, the medium was replaced and fluorescence microscopy was performed to detect the efficiency of transfection. The supernatants containing produced lentiviruses were collected every 24, 48, and 72 h after transfection.

### Lentivirus Concentration and Titration

In order to concentrate the produced lentiviruses, collected supernatants were filtered with 0.45-µm syringe filters, pooled and concentrated by PEG-8000. Subsequently, the vector titration was determined by adding different volumes including 1, 4, and 16 µL of concentrated viruses into 6×10^4^ Lenti-X 293T cells. Then, after 72 h, the viral titration was done by flow cytometry method based on the expressing green fluorescent protein (GFP) as transducing units per mL (TU/mL). All procedures were previously described (36).

### Transduction of NK-92 Cell Line

Briefly, NK-92 cells were seeded in 24-well plates at a density of 6×10^5^ cells in 1.5 mL complete Alpha MEM medium. Stimulation of NK-92 cells was done by human IL-2 (100 U/mL) 2 h before transduction. Concentrated virus particles with the MOI of 50 of the tittered virus were added to the medium-containing cells along with polybrene (8 μg/mL). To improve efficiency of transduction, centrifugation of NK-92 cells and virus-containing supernatant was done at 360 × g for 90 min at 32 °C. After 24 h, the medium was removed by spinning the cells at 360 × g for 5 min at room temperature (RT) and fresh complete medium was added. Finally, 72 h after the transduction, the transduced NK-92 cells were assessed by fluorescence microscopy and flow cytometry analysis. Transduced cells selected by incubation in medium containing 2 μg/mL of puromycin (Sigma-Aldrich, USA) and subsequently selected cells were cultured and expanded until to reach the acceptable density for next experiments.

### Flow Cytometry Analysis of Jurkat Cell Line

The expression level of CD25 on the cellular surface of Jurkat cell line was identified by flow cytometry. Approximately, 5×10^5^ Jurkat cells were washed three times and re-suspended in a total volume of 100 μL PBS. 0.125 μg of CD25 mAb-PE (BC96) (eBioscience, USA) and 0.5 μg of Mouse IgG1 kappa Isotype Control, PE (eBioscience, USA) were mixed, and the cells were kept for 30 min at RT and darkness. After washing with PBS, cells were evaluated with Cell Quest Pro software in a FACS Callibour (BD Biosciences, USA) instrument.

### PCR and Sequencing

The DNA extraction of the anti-CD25 CAR NK-92, mock and untransduced cells were done by Genomic DNA Isolation kit (from Tissue) (GeNet Bio, South Korea). Afterwards, the PCR reaction was as follows: 25 µL Taq DNA Polymerase 2× Master Mix Red with 2 mM MgCl_2_ (Amplicon, Denmark), 100 ng of template DNA and 10 pmol/µL of each primer including F: GACGACTTCGCCACCTACT and R: GTCCTCTTCACCTCCACCTT in a final volume of 50 µl. The amplification was performed on an Eppendorf Master cycler Gradient (Eppendorf AG, Hamburg, Germany) with the following program: 95 °C by 5′ for initial denaturation, 95 °C by 20′′, 58 °C by 30′′, 72 °C by 30′′ for 30 cycles and final extension at 72 °C by 5′. Also, the sequencing method of the anti-CD25 CAR construct was defined to assess the accuracy of the DNA sequence using the universal primers (CMV F-primer: CGCAAATGGGCGGTAGGCGTG and EBV R-primer: GTGGTTTGTCCAAACTCATC).

### RNA Isolation and qRT-PCR

According to the manufacturer’s protocol of NucleoSpin^®^RNA/Protein kit (Macherey-Nagel GmbH & Co.KG, Germany), the total RNA of the anti-CD25 CAR NK-92 cells was isolated. Based on the protocol, DNase I was utilized to remove the feasible contamination of the genomic DNA. Next, NanoDrop 2000C (Thermo Scientific, USA) was applied to determine the concentration of the isolated RNA. The first strand cDNA was synthesized by 2× RT Pre-Mix Kit (Biofact, South Korea) and its recommended protocol. The qRT-PCR primer sets pertained to the targeted transcript and the housekeeping gene were designed by AlleleID 7.7 software. Their sequences were contained as F CAR: GACGACTTCGCCACCTACT, R CAR: GTCCTCTTCACCTCCACCTT, F (GAPDH): CGGGAAGCTTGTGATCAATGG and R (GAPDH): GGCAGTGATGGCATGGACTG. Afterward, the expression of the anti-CD25 CAR was evaluated by qRT-PCR in triplicate for each sample as well as a non-template control (NTC) using Real-Time PCR Master Mix (2×) with high Rox reference dye (Biofact, South Korea) on Step-One Real-Time thermal cycler (ABI, USA). The reaction was contained 10 μL of 2× SYBR Green Master Mix, 10 pmol/μL of each primer and 1 μL of cDNA (50 ng/μL) in final volume of 20 μL. The program was carried out by initial denaturation at 95°C by 15′, followed by 40 cycles at 95°C by 20′′, 58°C by 35′′ and 72°C by 30′′ and final extension at 72°C by 3′. According to the melting curves produced by heating the amplicons from 55 to 95°C, the specificity of the amplifications was determined. Based on the 2^-ΔΔCt^ method, the relative expression of the anti-CD25 CAR was measured (37).

### Protein Isolation and Immunoblotting Analysis

The transmembrane protein of anti-CD25 CAR NK-92 cells was isolated by Mem-PER™ Plus Membrane Protein Extraction Kit (Thermo Scientific, USA). The presence of the protein of anti-CD25 CAR was confirmed by using 12.5% SDS-PAGE and silver nitrate staining method followed by Western blot analysis. For this purpose, all electrophoresed protein bands on 12.5% SDS-PAGE were transferred to a polyvinylidene difluoride (PVDF) membrane by a submarine method at 300 mA for 15 min. Next, for blocking the membrane, it was kept overnight at 4°C in 5% (w/v) skimmed milk. The membrane was rinsed three times with 0.05% Tween in PBS (PBS-T) and incubated by c-Myc mAb-HRP, clone: 9E10 (Invitrogen, USA) in the dilution of 1:1,000 at RT for 1.5 h. Finally, after three times-washing of the membrane by PBS-T, it was incubated in the mixture solution of clarity™ Western enhanced chemiluminescence (ECL) substrate (Bio-Rad, USA) for 2 min according to the manufacturer’s instruction. Then, the membrane was exposed to the X-ray film (Fuji, Japan) in darkness for 1 min and then, developed and fixed by the relative solutions for 1 and 5 min, respectively, to visualize the anti-CD25 CAR protein band.

### Antigen Binding Assay

An in-house ELISA assay was designed and accomplished for the verification of anti-CD25 CAR protein reactivity with human CD25 antigen. For this purpose, the CD25 antigen (Novus Biologicals, USA) with the final concentration of 8 μg/mL in 50 mM carbonate/bicarbonate buffer (pH=9.5) was added to each well of 96-well plate and kept at 4°C overnight. After blocking, 100 μL of the isolated anti-CD25 CAR protein with the concentration of 10 μg/mL was added into the test wells as well as the negative (no antigen) control wells. Meanwhile, 100 μL of the daclizumab (Novus Biologicals, USA) with the same concentration was added to the positive control wells and incubated at 37°C for 1 h. Then, the wells were washed three times with PBS. Subsequently, 100 μL of c-Myc mAb-HRP, clone: 9E10 (Invitrogen, USA) in the dilution of 1:750 was added to the test and the negative wells. In addition, 100 μL of Goat anti-mouse IgG (H+L)-HRP conjugate (Bio-Rad, USA) in the dilution of 1:1,000 was added to the positive control wells and incubated at 37°C for 1 h. After washing process, 100 μL of 3,3′,5,5′-tetramethylbenzidine (TMB) (Sigma-Aldrich, USA) was added to the wells and the plate was kept in the dark for 5 min. Finally, the reaction was stopped by using 2M sulfuric acid and the optical density (OD) was determined by a microplate reader (Hyperion, USA) at 450 nm.

### *In Vitro* Cytotoxicity Assay

The anti-CD25 CAR NK-92 cells and Jurkat cells were co-cultured at 37°C and 5% CO_2_ in a 24-well cell culture plate with an effector/target (E:T) ratio of 10:1 overnight. Then, cultured cells were harvested and prepared for staining. After co-incubation of target (Jurkat) cells together with effector (CAR NK) cells, total cell population was harvested. Staining was performed by Cell-Mediated Cytotoxicity Fluorometric Assay Kit (7-AAD/CFSE) (BD Biosciences, USA). Staining with CFSE and 7-AAD was performed based on manufacturer’s instruction. Briefly: 5 μL CFSE and 5 μL 7-AAD were added to the cultured cell and the cells were incubated for 15 min at RT in the dark. Finally, the cells viability was analyzed with flow cytometry method (FACS Calibur Becton Dickinson, USA).

The percentage of cytotoxicity was calculated using O¨ner O¨ zdemir et al. method (38). According to their formula, we used the percentage of CFSE positive/7-AAD-negative cells (viable cell population) for the target-cell gate in target-alone (control) and co-culture analysis, to correct for spontaneous apoptosis with the following formula:

Percent Cytotoxicity (PC)=([Control viable cellpercent]−[Co−incubation viable cellpercent])÷[Control viablecell percent]

### Interferon (IFN)-γ Release Assay

After an overnight co-culture of the anti-CD25 CAR NK-92 and Jurkat cells and untransduced NK-92 and Jurkat cells and also, single culture of anti-CD25 CAR NK-92 and untransduced NK-92 cells, the supernatants were collected by the centrifugation at 1,000 × *g* for 10 min at RT. Then, the ELISA assay was done for the detection of IFN-γ secretion in supernatants by Human IFN gamma ELISA kit (Invitrogen, USA), according to the manufacturer’s instructions.

### Statistical Analysis

Statistical analysis was carried out using SPSS 16.0 software (SPSS Inc, Chicago, USA), and comparisons among the groups were done by One-way ANOVA test. Results are shown as mean percentage ± standard deviation (SD), and p < 0.05 was considered as statistical significance.

## Results

### Modeling of the CD25 and Daclizumab scFv

Robetta web server predicted five best models for the daclizumab scFv. Subsequently, comparing the generated models showed that model 1 was more similar to the original protein structure (PDB ID: 3NFP). Thus, the selected model of scFv region was used for next steps. In addition, the missing loops in CD25 structure were fixed by SWISS-MODEL web server. PRISM web server predicted totally seven models for the interaction of the daclizumab scFv and CD25 based on the available complexes. PDB ID: 3IU3 was chosen by PRISM. By comparing the predicted complex models with similar crystal structures, model 1 with the energy score of −20.37 was more similar to the original PDB structure with PDB IDs: 3NFP and 3IU3 (**Figures S1–3**).

### Torsion Angles and ASA Results

Also, using VADAR version 1.8, RAMPAGE, Ramachandran plot, and Web-based PDB structural analysis service, the φ, ψ, and ω torsion angles of the scFv were completely calculated and the linker angles were separately mentioned in **Table S1**. Also, based on the evaluation of scFv residues, 233 residues (97.9%), four residues (1.7%), and one residue (0.4%) were placed in the favored, allowed, and outlier regions, respectively. RAMPAGE server showed that 98% and 2% of the residues expected in the favored and allowed regions, respectively. Also, ASA analysis demonstrated that the scFv possessed the proper surface area availability (**Figure S4**).

### Contact and Distance Maps of the Residues

NAPS showed that the scFv part had the regular structure in the extracellular region. Particularly, the distance and contact maps of the scFv were in normal ranges (**Figures S5**, **6**).

### MD Simulation

**Figure 2:**
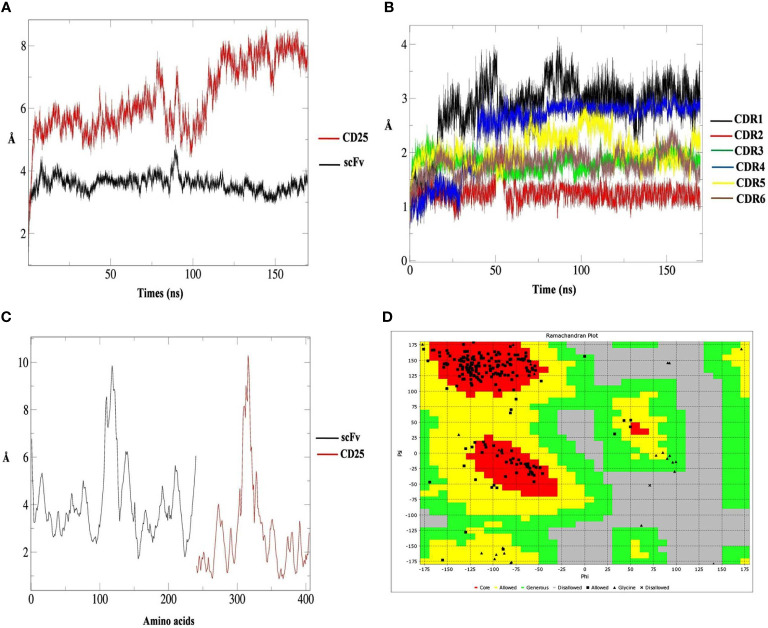
The root mean square deviation (RMSD) results of CD25 (red graph) and scFv (black graph) in predicted interaction model during 170 ns analysis **(A)**, the RMSD results of scFv CDR1 (black graph), CDR2 (red graph), CDR3 (green graph), CDR4 (blue graph), CDR5 (yellow graph) and CDR6 (brown graph) **(B)**, the RMSF results of scFv (black graph) and CD25 (red graph) **(C)** and the Ramachandran plot for the scFv containing the (Gly_4_Ser)3 linker, the core parts (red), allowed parts (yellow), generous parts (green), disallowed parts (grey), allowed residue (black square), glycine (black triangle), and disallowed residue (black multiplication sign) **(D)**.

The RMSD values of scFv showed a stable structure and no remarkable structural changes during 170 ns. However, the RMSD value for CD25 structure demonstrated some changes during the simulation (**Figure 2A**). RMSD graphs of six CDRs, showed that CDR2 had no structural changes except for the negligible changes and the CDR1 had the highest structural changes during 170 ns (**Figure 2B**). According to the RMSF graphs of six scFv CDR regions, it was shown that these regions had low flexibility compared to other parts of the scFv and the (Gly_4_Ser)3 linker had a higher flexibility value (**Figure 2C**). Ramachandran plot indicated that most of the torsions are located at allowed region (**Figure 2D**). Based on the H-bond calculations, it was shown that the lowest hydrogen bonds were related to CDR1 and CDR6 of scFv with CD25 and the highest hydrogen bonds were observed for CDR4 and CDR5 with CD25 over 40,000 frames of simulation (**Figures S7–12**). These results showed that the six CDRs were engaged in CD25 binding sites and scFv-CD25 complex had the proper bindings pose with desirable amounts hydrogen bonds.

### Generation of NK-92 Cell Line Expressing Anti-CD25 CAR

**Figure 3:**
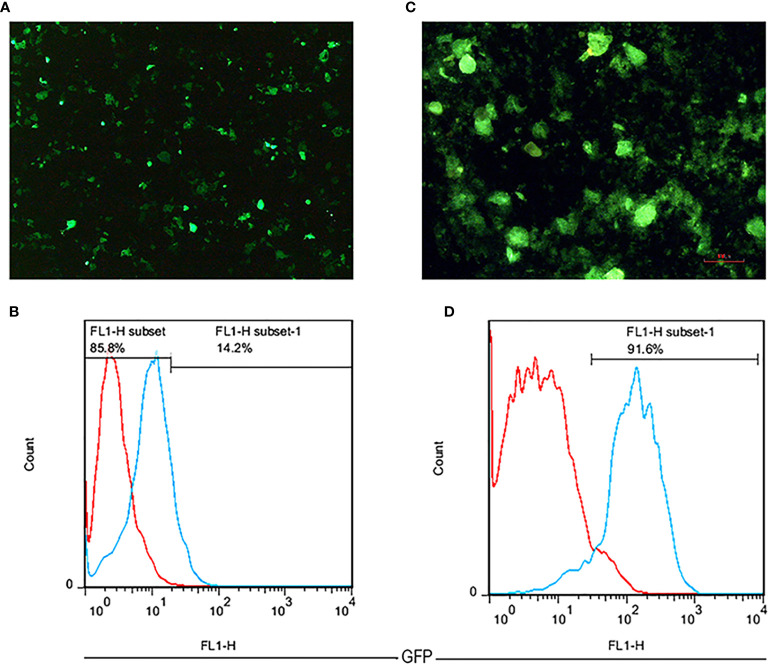
The fluorescent microscopic image of transfected Lenti-X 293T cells with 40× magnification **(A)**. A flow cytometry histogram chart of the percentage of GFP expression in transfected Lenti-X 293T cells expressing anti-CD25 CAR **(B)**. The fluorescent microscopic image of transduced NK-92 cells with 100 × magnification **(C)**. A flow cytometry histogram chart of the percentage of GFP expression in transduced NK-92 cells expressing anti-CD25 CAR **(D)**.

Following the three plasmids co-transfection, GFP expressing Lenti-X 293T cells were observed as brilliant green shining cells after 16 h under a fluorescence microscope. The high rate of green shimmer indicated the high efficiency of transfection (**Figure 3A**). To determine the titration of the produced lentiviruses, the transduced Lenti-X 293T cells were analyzed by flow cytometry analysis. It was shown that the GFP positive cells percentage expressing anti-CD25 CAR was 14.2% using 16 µl of viruses (**Figure 3B**). Based on the MOI=50, the given volume of the concentrated viral supernatants transduced NK-92 cell line at the rate of 91.6%. It proved by fluorescence microscopy (**Figures 3C, D**) and by the resistance to the given final concentration of puromycin.

### CD25 Expression on the Cell Surface of Jurkat Cell Line

**Figure 4:**
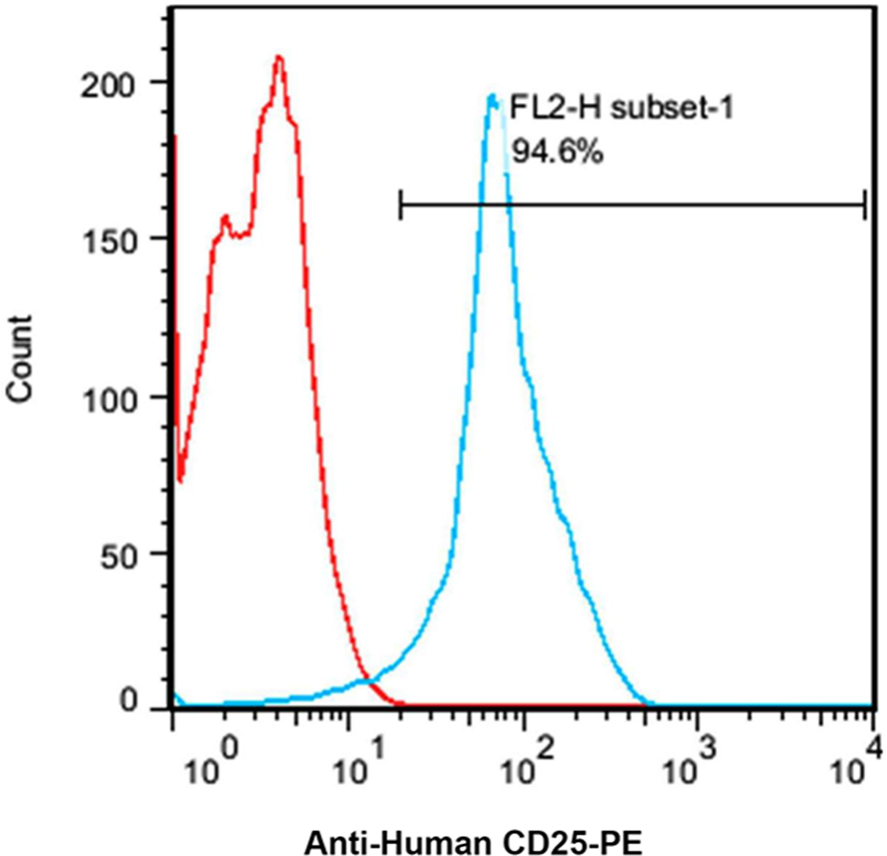
A flow cytometry histogram chart of the percentage of CD25 expressing Jurkat cells. About 95% of the Jurkat cells express CD25 as indicated by anti-human CD25-PE antibody.

Based on the flow cytometry results, the expression level of CD25 on the cellular surface of Jurkat cell line was about 94.6% (**Figure 4**).

### PCR and Sequencing of the CAR Construct

**Figure 5:**
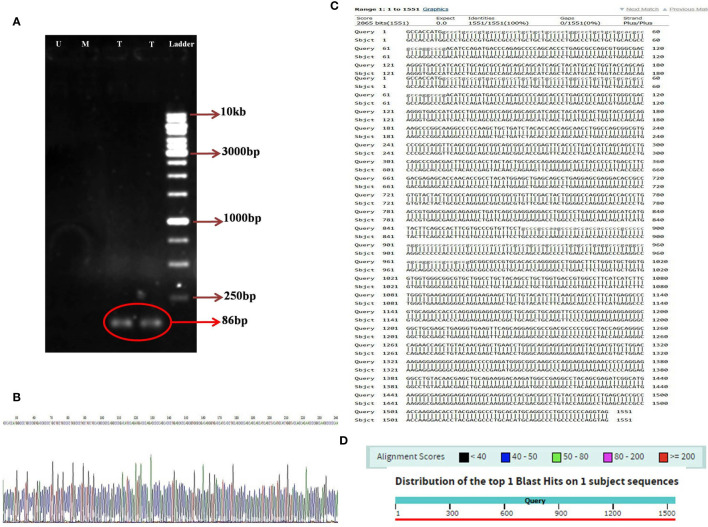
The image of electrophoresed PCR product on 1% TBE agarose gel **(A)**; DNA ladder (Thermo Fisher Scientific, USA) with the Cat. Number of SM1163 (Ladder), two lanes relative to amplified 86 bp specific band in transduced NK-92 cells containing anti-CD25 CAR construct (T), one lane relative to unamplified specific band in transduced mock NK-92 cells (M), one lane relative to unamplified specific band in untransduced NK-92 cells (U), the sequencing result of anti-CD25 CAR construct. A part of the sequencing result is displayed **(B)**. The nucleotide alignment result of anti-CD25 CAR construct using nucleotide BLAST of NCBI web server **(C)**. The graphic result of the nucleotide alignment result of anti-CD25 CAR construct using nucleotide BLAST of NCBI web server **(D)**.

Utilizing the specific primers, the 86 bp PCR product was amplified from the variable light chain of the anti-CD25 CAR NK-92 cells. However, the mock and untransduced cells had no the amplified fragment (**Figure 5A**). In addition, the sequencing results of the anti-CD25 CAR construct demonstrated that the designed CAR possessed the accurate and precise DNA sequence compared with the nucleotide alignment (**Figures 5B–D**). Also, the Kozak sequence, codon optimization, reading frame and stop codon were well defined.

### Expression Level of the Anti-CD25 CAR

**Figure 6:**
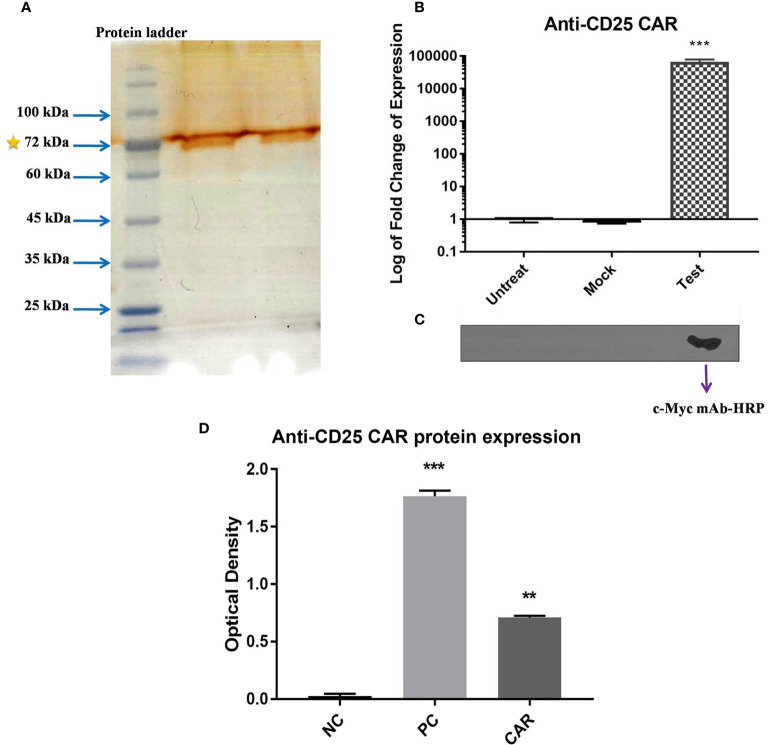
Confirmation of the expression of CAR on NK-92 cells. SDS-PAGE method revealed a 70 kD band representing the expression of anti-CD25 CAR protein **(A)**. The bar chart represents the significant increase in anti-CD25 CAR transcript level of test group compared with mock and untreated group cells (p < 0.0001) using qRT-PCR method **(B)**. Confirmation of the presence of anti-CD25 CAR proteins among isolated membrane proteins using the anti c-Myc mAb-HRP in western blotting and X-ray film in ECL method **(C)**. The transduced NK-92 cells with anti-CD25 CAR construct (Test) compared with (Mock) and untreated cells (Untreat) shows that only one band is appeared in test group and there is no band for other groups. The ELISA bar chart confirms the binding of anti-CD25 CAR proteins to human CD25 antigen **(D)**. The bar charts of NC, PC, and CAR are considered as negative control, positive control (daclizumab), and anti-CD25 CAR, respectively. The asterisk sign indicates the statistical significance (***p = 0.0001 and **p = 0.001).

The anti-CD25 CAR expression was verified with qRT-PCR, SDS-PAGE, and Western blot analyses. A sharp band was visualized with the approximate molecular weight of 70 kDa on the SDS-PAGE (**Figure 6A**). As illustrated in **Figure 6B**, mRNA expression levels of anti-CD25 CAR were evaluated with qRT-PCR in transduced NK-92 cells by pCDH-513B-1-anti-CD25 CAR vector, as well as in transduced NK-92 cells by mock vector, and untransduced NK-92 cells (negative control). The results showed that the level of the anti-CD25 CAR mRNA in transduced group was significantly higher than the negative control and the mock groups (p < 0.0001). However, Western blotting was needed to confirm the presence of anti-CD25 CAR proteins among other isolated membrane proteins by specific antibody. Western blotting method using the anti c-Myc mAb-HRP revealed a single and specific band on an X-ray film pertained to the 70 kDa band (**Figure 6C**).

The anti-CD25 CAR protein’s molecular weight was approximately calculated as a 53.94 kDa protein by Protein Molecular Weight web server (https://www.bioinformatics.org/sms/prot_mw.html) without considering the glycosylation (**Figure S13**). It is noteworthy that proteins undergo post-translational modifications such as glycosylation in eukaryotic cells. Two major types of glycosylation include N-linked and O-linked glycosylations. The obtained data from NetNGlyc 1.0 Server and NetOGlyc 4.0 Server showed that the anti-CD25 CAR protein had 10 predicted N-linked glycosylation sites and seven predicted O-linked glycosylation sites (**Figures S14**, **15**). The NetNGlyc 1.0 Server identified three sites with the highest probability of N-glycosylation. One site was Asn177 with its usual arrangement of adjacent residues, Asn-X-Ser/Thr. Two other sites included Asn403 and Asn441. Usually, the heaviest N-linked glycosylation arrangement weighs 2.7 kDa (39). Considering these three predicted N-linked glycosylation sites in the anti-CD25 CAR protein, approximately 8.1 kDa was added to its molecular weight and weighed approximately 62 kDa. The molecular weight of the O-linked glycosylation arrangement is much lower than that of the N-linked glycosylation; usually its heaviest arrangement weighs 1 kDa (40). In this regard, considering seven predicted O-linked glycosylation sites, the molecular weight of the anti-CD25 CAR protein can be estimated as about 69 kDa which is very close to the estimated molecular weight appeared on the SDS-PAGE.

### Confirmation of Anti-CD25 CAR Reactivity with CD25 Antigen

The reactivity of the anti-CD25 CAR protein with human CD25 antigen was confirmed by ELISA method. The optical density (OD) of the test and negative control groups were 0.7 ± 0.02 and 0.01 ± 002, respectively, and their difference was highly significant (p = 0.0001). The 70-fold difference between the test and negative control groups pointed out the strong interaction of the anti-CD25 CAR and the human CD25 antigen. The OD of the positive control (daclizumab) was 1.731 ± 0.08, exhibiting 2.47-fold increase in comparison to the test (p = 0.002) (**Figure 6D**). In addition, affinity of the anti-CD25 scFv (the binding unit of the CAR) was determined using ELISA method and Beaty equation, and was calculated as 5.01×10^-7^ M (data in press).

### Efficacy of Anti-CD25 CAR NK Cells Cytotoxicity and IFN-γ Production Against CD25^+^ Jurkat Cells

**Figure 7:**
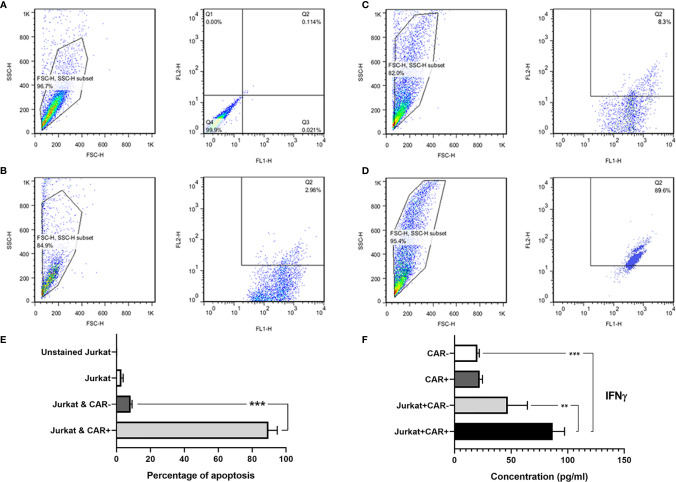
The flow cytometric dot-plot graphs of *in vitro* cytotoxicity assay and IFNγ secretion level measured with ELISA method. Unstained single culture of Jurkat cells **(A)**; single culture of Jurkat cells stained with 7-AAD **(B)**; Jurkat cells co-cultured with NK-92 cells showing only 8.3% cytotoxicity **(C)**; and Jurkat cells co-cultured with anti-CD25 CAR expressing NK-92 cells showing 89.6% cytotoxicity **(D)**. FL1: CFSE, and FL2: 7-AAD. The bar chart shows the highest amount of apoptosis in Jurkat cells co-cultured with anti-CD25 CAR expressing NK-92 cells in comparison with other groups **(E)**. As depicted in the bar chart, the CAR NK cells co-cultured with the target cells (Target/CAR+ NK) produced the highest level of IFNγ **(F)**. The asterisk sign indicates the statistical significance (***p = 0.0001 and **p = 0.001).

The anti-CD25 CAR NK-92 cells efficiently and specifically lysed the Jurkat cells at E:T ratio of 10:1 after overnight incubation. In fact, 89.6% of the target cells were lysed. By contrast, un-equipped NK-92 cells had no palpable cytotoxicity (8.3%) (**Figures 7A–D**) and the difference was significant (p = 0.0001) (**Figure 7E**). In order to evaluate probable fratricide effect, we carried out a flow cytometric analysis on the CAR-equipped NK-92 cells for expressing CD25. The results showed only 0.11% CD25 expression on CAR NK-92 cells (**Figure S16**). As this is an almost zero expression, there is no fratricide phenomenon and the cytotoxicity is happened on Jurkat cells.

The co-culture of anti-CD25 CAR NK-92/Jurkat cells produced significantly a higher level of IFN-γ compared with the co-culture of untransduced NK-92/Jurkat cells (86.72 pg/mL *vs* 47.01 pg/mL; p = 0.006). In addition, the level of IFN-γ secretion by anti-CD25 CAR NK-92/Jurkat cells co-culture was significantly higher than the culture of untransduced NK-92 cells alone (86.72 pg/mL *vs* 20.3 pg/mL; p = 0.0002). Meanwhile, there was no significant difference between IFN-γ production by anti-CD25 CAR NK-92 and untransduced NK-92 cells (22.26 and 20.3 pg/mL; p = 0.995) cultured in the absence of the target cell (**Figure 7F**).

## Discussion

Genetic manipulation of T cells and NK cells to express an anti-tumor antigen has emerged as a promising immunotherapeutic strategy for different malignancies. The major prerequisite for developing CAR-expressing immune cell against malignancies is to identify an appropriate target antigen (32). Treg cells (FOXP3^+^Tregs) along with myeloid-derived suppressor cells (MDSCs) and tumor-associated macrophages (TAMs) type 2, perform immunosuppressive functions by their high level accumulations in TME (14). Among these cells, Tregs play a highlighted role in cancer ignorance, cancer immunologic tolerance and cancer poor prognosis by (a) secretion of immunosuppressive agents including TGF-β, IL-10, and IL-35, (b) Perforin/Granzyme-mediated direct cytotoxicity, (c) metabolic interruption by consumption of IL-2 and making IL-2 deprivation in TME, cAMP- and A2 adenosine receptor-mediated immunosuppression, (d) dendritic cells (DCs) suppression by cell-cell interaction, and (e) inhibitory receptors and immune checkpoints including IDO, ICOS, CTLA-4, TIM-3, LAG-3, PD-1, and TIGIT (15). Therefore, Treg cells can hamper cancer treatment by infiltrating tumors and hindering antitumor immune responses of tumor antigen-specified CD8^+^ T cells and CD4^+^ T cells (16).

Although, there are some other immune cells expressing CD25 including CD25^+^ B cells (41), activated T cells (2, 3), NK cells (42), and myeloid DCs (43), CD25 is considered to function a more significant role in immune tolerance (44, 45). This happens since CD25 is expressed transiently on activated effector cells and constitutively on Treg cells. Tregs rely on IL-2 signaling and constitutive expression of CD25 on Tregs is critical for their survival (46–48).

Treg cells are the main obstacle in the way of reaching a successful cancer treatment. Indeed, decreased CD8^+^ T to Treg cells ratio is correlated with a poor prognosis, unfavorable treatment outcome and finally diminished survival of patients suffering from various types of cancers such as non-Hodgkin’s lymphoma (NHL) (49), ovarian cancer (50), lung cancer (51), glioblastoma (52), pancreatic ductal adenocarcinoma (53), breast (54), hepatocellular carcinoma (55), cervical cancer (56), head and neck cancer (57), gastric cancer (58), melanoma and other malignancies (59). In acute myeloid leukemia (AML), but not in NHL, leukemic blast cells elevate the frequency of Tregs in TME (60). Accordingly, it has been reported that the higher levels of Tregs could be useful as an early diagnostic marker for timely diagnosis of chronic lymphocytic leukemia (CLL) at the initial steps (61). In some advanced cancers, it has been demonstrated that there are accumulating and infiltrating CD4^+^CD25^high^ T cells surrounding tumors (62). In other types of cancers, like colorectal cancer, CD4^+^CD25^+^FOXP3^+^ Tregs are extensively increased in tumors in correlation with their stages (63, 64). Onizuka et al.** and Shimizu et al. have suggested that the eradication of CD4^+^CD25^+^ Tregs by anti-CD25 mAb can improve the egress of various tumors *in vivo* and *in vitro* (22, 65). Accordingly, targeting and eliminating of the Tregs has been considered as a promising treatment for AML and ALL (66, 67). On the basis of all these findings, the idea of targeting Tregs using a CAR NK cell rather than a mAb provoked us to speculate that CD25 might be an ideal antigen. In the current study, utilizing anti-CD25 CAR-modified NK-92 cells, we managed to develop a novel and potentially promising strategy against Tregs as the major immune regulatory element in TME.

Generally, CAR NK cells are based on NK-92 cell line, primary cord blood (CB)-derived, and peripheral blood (PB) NK cells. They have been entered into clinical trials examining the efficacy in different phases (68–71). Remarkably, the transformed cell line NK-92 was originated from undifferentiated NK cell precursors (72–74). This malignant origin of NK-92 led to a barrier in the advancement of NK-92-based cell therapy but it was overcame by irradiation treatment (75, 76). The infusion of irradiated unmodified NK-92 cells have been demonstrated to be safe in patients suffering from malignancies (77). In spite of a proliferation-limiting irradiation, scientists have notably illustrated *in vitro* and *in vivo* cytotoxic activity of CAR-engineered NK-92 cells. Limiting amounts of isolated primary NK cells (only 10% of all lymphocytes) has been shown as a challenge in the development of NK cell-based therapies, hence, the cell enrichment is needed (selection for CD56^+^ cells) in autologous usage and MHC-matched donor or depletion of alloreactive T cells to prevent GVH reactions in allogeneic donor is critical (78, 79). On the other hand, NK-92 cells can be simply expanded in serum free-medium without feeders, but IL-2 is merely needed (in flasks, bags, or bioreactors). Primary NK cells need engineered feeders (for instance, K562 cells expressing IL-15 and TNFSF9) together with IL-2 in the corresponding situations. NK-92 cells, as the pure cell line, have been able to be engineered at high efficiency rates. In contrast, primary NK cells have not been able to be engineered at these rates. Both NK-92 and primary NK cells possess limited life span in recipient patients, whereas the proliferation of NK-92 cells has been almost terminated by irradiation, as above mentioned. Also, there are no concerns about persisting CAR-associated side effects by both primary NK and NK-92 cells. Both of them have no need for an extra suicide gene construct. There is feasible to keep NK-92 cells by cryopreservation process and also expansion of this cell line upon thawing before infusion. This is possible to have donor NK cells cryopreserved, however, recovery is poor upon thawing (79). Here, a third generation construct of CAR NK-92 against CD25, with the prospect of overcoming immune escape mechanism in cancers, was designed and the corresponding protein product was evaluated using bioinformatics methods. From the viewpoint of the protein structure, this work was mainly focused on the scFv as the binding part of the anti-CD25 CAR. For this aim, a few models were predicted based on the available PDB structures. Then, the predicted models further analyzed by structural alignment for the interaction of scFv and CD25. It was shown that the model 1 was considerably similar to the crystallographic structure and the calculated energy score for the scFv and CD25 complex was -20.37. Then, molecular dynamics simulation was performed for 170 ns. Based on the MD results, the RMSD values showed that the scFv had a stable structure and no remarkable structural changes were observed during 170 ns. However, the RMSD value for CD25 structure showed considerable changes during the simulation. Evaluating the structural changes of CD25 showed that residues 65-101 had the highest RMSD value which was affected the overall RMSD graph for CD25. It is worth to mention that this region was the missing part of CD25 which was not resolved in the crystal structure and it seems that our results were in accordance with the experimental studies. Also, there were other small breaks found in the CD25 structure including residues 31-35, 131-138, and 142. Based on the RMSF graphs for six CDR regions of scFv it was shown that these regions had low flexibility compared to other parts of the scFv. Queen et al. reported that these six CDR regions engaged possibly in binding sites of CD25 (80). In their study, the highest structural flexibility of scFv was related to the (Gly_4_Ser)3 linker including residues 111-125. Based on the Chen et al., the (Gly_4_Ser)3 linker has been categorized as the flexible linker (81) and this feature was observed in the RMSF graph of scFv. RMSF results showed that the structural flexibility of CD25 was at normal ranges except for the fixed loop (residues 65-101) which had the highest amount of flexibility throughout the CD25 structure. According to the RMSD graphs for six CDR regions of scFv (including CDR1: Arg24 to Ala34, CDR2: Lys50 to Ser56, CDR3: Gln89 to Met97, CDR4: Arg153 to Ile157, CDR5: Gly172 to Gly188, and CDR6: Gly221 to Glu228), it was shown that CDR1 and CDR4 had higher RMSD values compared with other CDRs. Nearly, the CDR2 had no structural changes except for the negligible changes over the simulation time. The CDR1 had the highest structural changes during 170 ns. Also, the numbers of hydrogen bonds during the simulation for 40,000 frames were analyzed. The lowest hydrogen bonds were related to CDR1 and CDR6 of scFv and the highest hydrogen bonds were pertained to CDR4 and CDR5 of scFv. Indeed, the numbers of hydrogen bonds from the highest to lowest were calculated in CDR5, CDR4, CDR3, CDR2, CDR1, and CDR6, respectively. These results showed that the six CDRs were engaged in CD25 binding sites and scFv and CD25 had the proper bindings pose with desirable amounts hydrogen bonds. Furthermore, the φ, ψ, and ω torsion angles of the most residues (99.6%) were placed in the favored and allowed regions, specifically, the angles for scFv linker were appropriate. ASA analysis illustrated that anti-CD25 CAR protein structure had the regular structure. In addition, the anti-CD25 CAR protein possessed the normal binding pose in the scFv region. Also, the (Gly_4_Ser)3 linker had the positive effect on the scFv structure. Structural analyses showed that the scFv with (Gly_4_Ser)3 linker was able to bind to CD25 target with the appropriate binding pose. Additionally, the scFv with (Gly_4_Ser)3 linker possessed the proper flexibility between variable light and heavy chain to target and bind to CD25. Thus, the scFv with (Gly_4_Ser)3 linker, as the functional targeting area of the anti-CD25 CAR, might be reliable to apply for the CAR structure. It was proposed that this linker might be applied for designing the different types of CAR molecules requiring to the flexibility to target the different antigens.

In fact, there are a few reports that focused on the computational analyses of CARs. For example, Pirooznia et al. have used the molecular dynamics simulation to investigate the impact of the presence of spacers on conformational changes in the binding sites of single variable domain on a heavy chain (VHH) in CAR T cell. They explained that the presence of spacer FcγIIα has led to an enhancing influence on VHH with MUC1 interaction (82). Hegde et al. have applied the docking method to survey the tandem CAR (TanCAR) T cell targeting HER2 and IL13Rα2 in glioblastoma. They found the arrangement of the TanCAR domains has simultaneously permitted targeting both receptors. They concluded that the considered flexible linker between the domains has possibly allowed the conformational variability of domain arrangement, so permitting the IL-13 mutein and the FRP5-scFv to optimize the simultaneous targeting of the two receptors (83). In fact, *in silico* methods such as molecular modeling and simulation can be invaluable and helpful ways for predicting the interactions and dynamics of the significant parts of CARs like scFvs. Although various computational tools do not have absolute accuracy, however, they can relatively provide precise predictions of events at the RNA and protein levels. Therefore, using these tools is recommended to analyze the performance of different types of CAR molecules to reduce the cost and the time of the research.

In order to characterize the present CAR NK cell and to confirm its functionality, several experimental assays were done. The generation of transduced NK-92 cells with the anti-CD25 CAR construct was initially proved by PCR and sequencing in comparison with the untreated and mock cells. The expression level of the anti-CD25 CAR mRNA was significantly higher than the negative control and mock cells (p < 0.0001). The identity of the CAR protein was confirmed with the SDS-PAGE and Western blotting methods. The reactivity of the anti-CD25 CAR protein to human CD25 antigen compared with the positive control (daclizumab) and negative control was confirmed by ELISA method. The reactivity of the CAR was lower than the mAb, daclizumab, (p = 0.002), while it was significantly higher than the negative control and showed a tangible degree of reactivity against CD25 protein (p = 0.0001). Regarding to the fact that CAR contains only one antigen binding capacity, it is reasonable to show lower reactivity to antigen when compared with a bivalent mAb.

As another functional assay, the co-culture of the anti-CD25 CAR NK-92 and Jurkat cells proved that the NK-92 cells expressingthe anti-CD25 CAR were capable of lysing 89.6% of the target cells. Regardless of the fact that based on datasheet presented in ATCC company web site (https://www.atcc.org/products/all/CRL-2407.aspx#characteristics) the NK-92 cell line does not express CD25 marker, we carried out a flow cytometric analysis on our CAR-equipped NK-92 cell to evaluate the probable fratricide effect. The results showed only 0.11% CD25 expression on the CAR NK-92 cells. As the expression of CD25 on the CAR NK-92 cells was almost zero, no fratricide phenomenon is expecting. Maki et al. have reported that, the expression status of CD25 in NK-92 cells is not clear and they showed that the expression of CD25 onactivated NK-92 cells after treatment with 100 IU/mL IL-2 is low (73). Gong et al. have shown that in the presence of 100 IU/mL of IL-2 about half of NK-92 cell line can express CD25. They also showed the expression of CD25 on the cell line is inversely correlated with IL-2 concentration (Gong et al., 1994). This is rational as when there is adequate amount of IL-2 accessible for NK cells, especially for long time, there is no need to express CD25. Although NK-92 cell is an IL-2 dependent cell but this is related to IL-2R βγc complexes rather than CD25, as expected and as reported by Gong et al. Thus, NK cells try to down-regulate CD25 because only the expressed IL-2R βγc complex is enough for the cells that use other cytokines like IL-15, IL-21, and IL-18 as the most important growth factors (84, 85). Based on this, it seems that CD25 expression on NK-92 cells, if any, could normally been down-regulated as time goes by and our CAR-equipped NK-92 cells may have lost their CD25 expression after about a couple of weeks of culture in the continuous presence of 200 U/mL IL-2 in our experiments.

In addition, using ELISA method for IFN-γ secretion in the cell co-culture situation, it was represented that the anti-CD25 CAR NK-92 cells produced and secretedthe highest amount of IFN-γ when cultured with CD25 bearing Jurkat cells compared with the co-culture of untransduced NK-92 and Jurkat cells, as well as other conditions. Upon binding of the CAR NK cells to their targets, this cytokine is highly released by these engineered NK cells. Our findings were in accordance with other reports of CAR NK cells against different target cells (32, 33, 86, 87).

In spite of the progressions in targeting tumor antigens, problems related to the success of cancer treatment such as treatment failure and cancer immune escape leading to cancer relapse have yet been unresolved. Based on this, by constructing and evaluating of the anti-CD25 CAR NK cells, we tried to target a prominent marker expressed on the most important regulatory factors, Treg cells, in TME. Up to our knowledge, this is the first work investigating the potential of CAR immune cells to target CD25 marker. It seems that the removal of Tregs from TME has high therapeutic potential benefitsfor most types of cancers. However, a potential challenge of targeting CD25^+^ Tregs by this CAR NK cell is the probability of killing CD25^+^ activated effector T cells which are necessary for eradication of tumors. It has been shown that depletion of Tregs in animal models leads to increased anti-tumor immunity. Moreover, a pre-clinical study has demonstrated that the depletion of Tregs prior to immunization with a cancer vaccine comprising dendritic cells presenting carcinoembryonic antigen can increase the antigen-specific T cell responses (4). Based on a phase I clinical trial outcomes, infusion of daclizumab in patients with metastatic breast cancer was associated with a noticeable and prolonged removal of CD25^+^ FOXP3^+^ Tregs in circulation. Administration of a cancer antigen peptide vaccine during this period resulted in effective generation of cytotoxic T cells against the tumor antigen (6). Therefore, long-term CD25 blockade or elimination of Tregs provide an approach to overcome a main element of immune suppression in cancer patients (5). These also might be correct for the presented anti-CD25 CAR NK cell and more potent and prolonged Treg-elimination effect would be expected *in vivo*. In addition, it is known that activated T cells, as well as activated monocytes and macrophages secrete soluble CD25 (sCD25) that competes with membrane-bound CD25, and hence by skewing IL-2 signaling from high affinity toward intermediate affinity can increase T cell survival, leading to more efficient expansion of activated T cells (1, 88). Theoretically, the presented anti-CD25 CAR NK cell can lyse numerous CD25^+^ cells and hence, high amount of sCD25 will be released which in turn, can improve adaptive immune responses. Nevertheless, further studies are required to support this hypothesis.

On the other hand, CD25 is expressed by the malignant cells of a number of cancers such as adult T-cell leukemia/lymphoma, cutaneous T-cell lymphomas, hairy cell leukemia, anaplastic large-cell lymphoma, granulocytic neoplasms and the Reed-Sternberg cells and associated polyclonal T cells in Hodgkin’s lymphoma. Although daclizumab has FDA approval for use in organ transplantation, it has also been demonstrated that applying daclizumab in clinical trials are providing an effective therapeutic approach for patients with CD25 expressing leukemia and lymphoma (3, 12, 89, 90). However, it would be of great value to develop another therapeutic tool with enhanced antitumor efficacy than daclizumab. Therefore, the CAR NK cell presented in this work can be a promising candidate.

In conclusion, we developeda novel anti-CD25 CAR-expressing NK-92 cells to target CD25^+^ T cells and demonstrated that the expression of this CAR on NK-92 cells dramatically enhanced their cytolytic activity and IFN-γ production when co-cultured with CD25^+^ Jurkat cells *in vitro*. Like other pharmaceutical agents, this proposed anti-CD25 CAR may have side effects including the reduction of Treg cells and onset of autoimmunity as well as some degree of off-target effect. With the aim of finding its efficacy against various solid and liquid cancers and its side effects, *in vivo* analyses must be carried out to evaluate the pros and cons of anti-CD25 CAR in animal bodies. This work prepared a proper and functional CAR NK cell for further *in vitro* and *in vivo* studies, with the perspective of clinical applications, either applied alone or in combination with other therapeutic approaches for the treatment of different types of cancer and other relevant pathologic conditions.

## Data Availability Statement

The original contributions presented in the study are included in the article/**Supplementary Material**. 

## Author Contributions

Experimental performances: MD. Bioinformatic analyses: MD and MRG. Study design: MD, ZH, MG-H, AS, WC, and MM-B. Study conduct: ZH, MG-H, AS, and MM-B. Data collection: MD. Experimental data analyses: MD and MG-H. Data interpretation: MD, ZH, MG-H, MRG, WC, AS, and PN. Drafting manuscript: MD. Revising manuscript content: ZH, WC, and MG-H. Approving final version of manuscript: MD, ZH, WC, and MG-H. All authors contributed to the article and approved the submitted version.

## Funding

The project was financially supported by The Graduate Office of University of Isfahan (Grant No. 8489).

## Conflict of Interest

The authors declare that the research was conducted in the absence of any commercial or financial relationships that could be construed as a potential conflict of interest.
