# Supplementary material for: A Novel CAR Expressing NK Cell Targeting CD25 With the Prospect of Overcoming Immune Escape Mechanism in Cancers
Source: Front Oncol. 2021 May 14;11:649710. doi: 10.3389/fonc.2021.649710 (PMC8160382; doi:10.3389/fonc.2021.649710)
Supplement: Supplementary file 1 [file DataSheet_1.docx]

**Supplementary files**

**Figure S1.** The predicted models by PRISM web server. The first model with energy of -20.37kcal/mol was selected for further analysis.

**
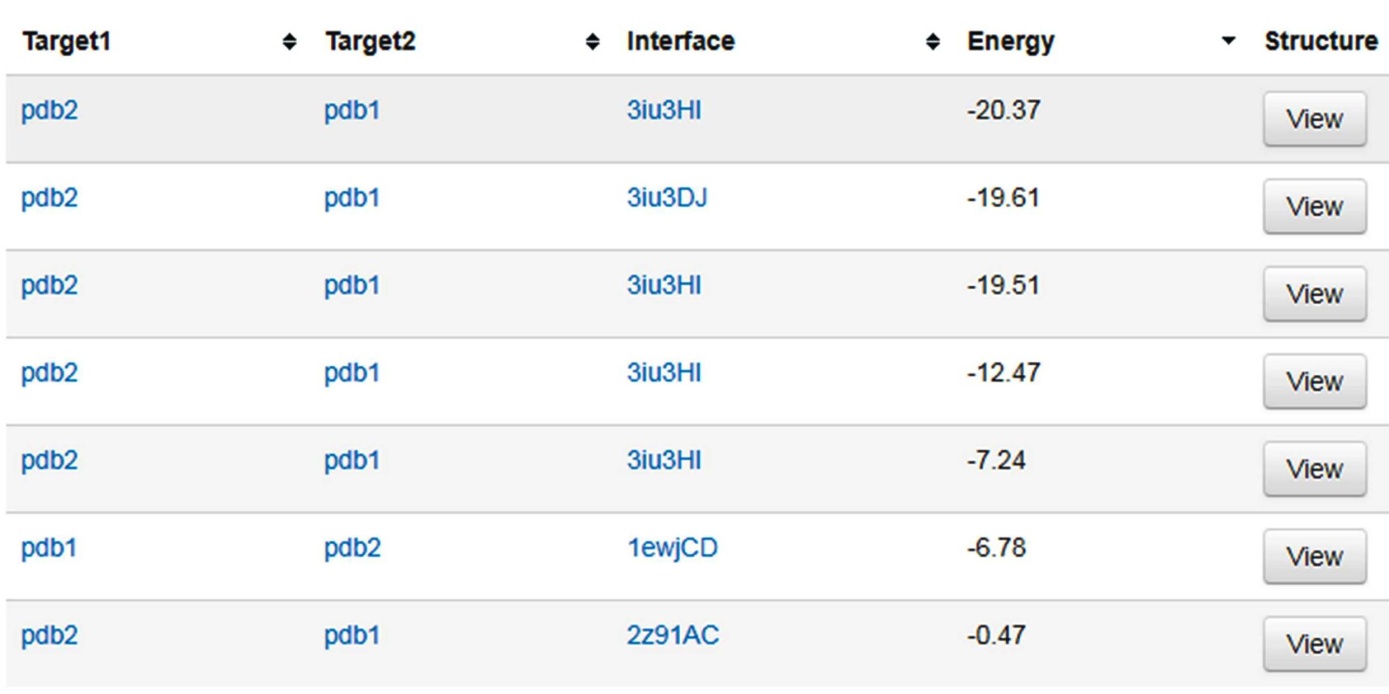
**

**Figure S2.** The “model 1” with energy of -20.37kcal/mol obtained from PRISM showed the similarity with the original PDB IDs: 3IU3 and 3NFP. The amino acids including Leu42, Tyr43, and Met25 in this model pertained to CD25 structure (as illustrated in PDB ID: 1Z92) are the critical amino acids for CD25/IL-2 binding.

**
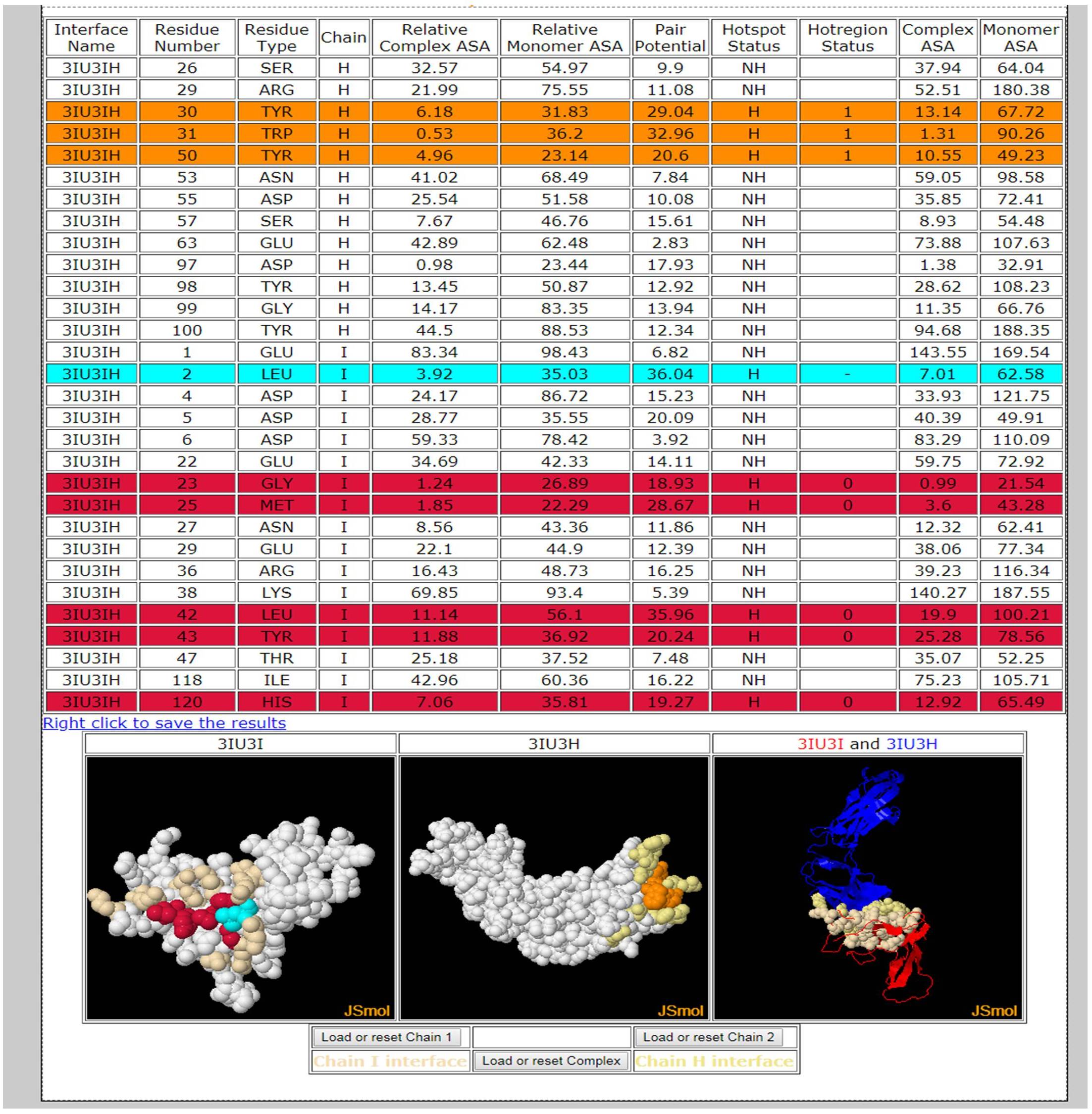
**

**Figure S3.** The interface residues contacts were listed below for the “model 1” by PRISM web server.

**
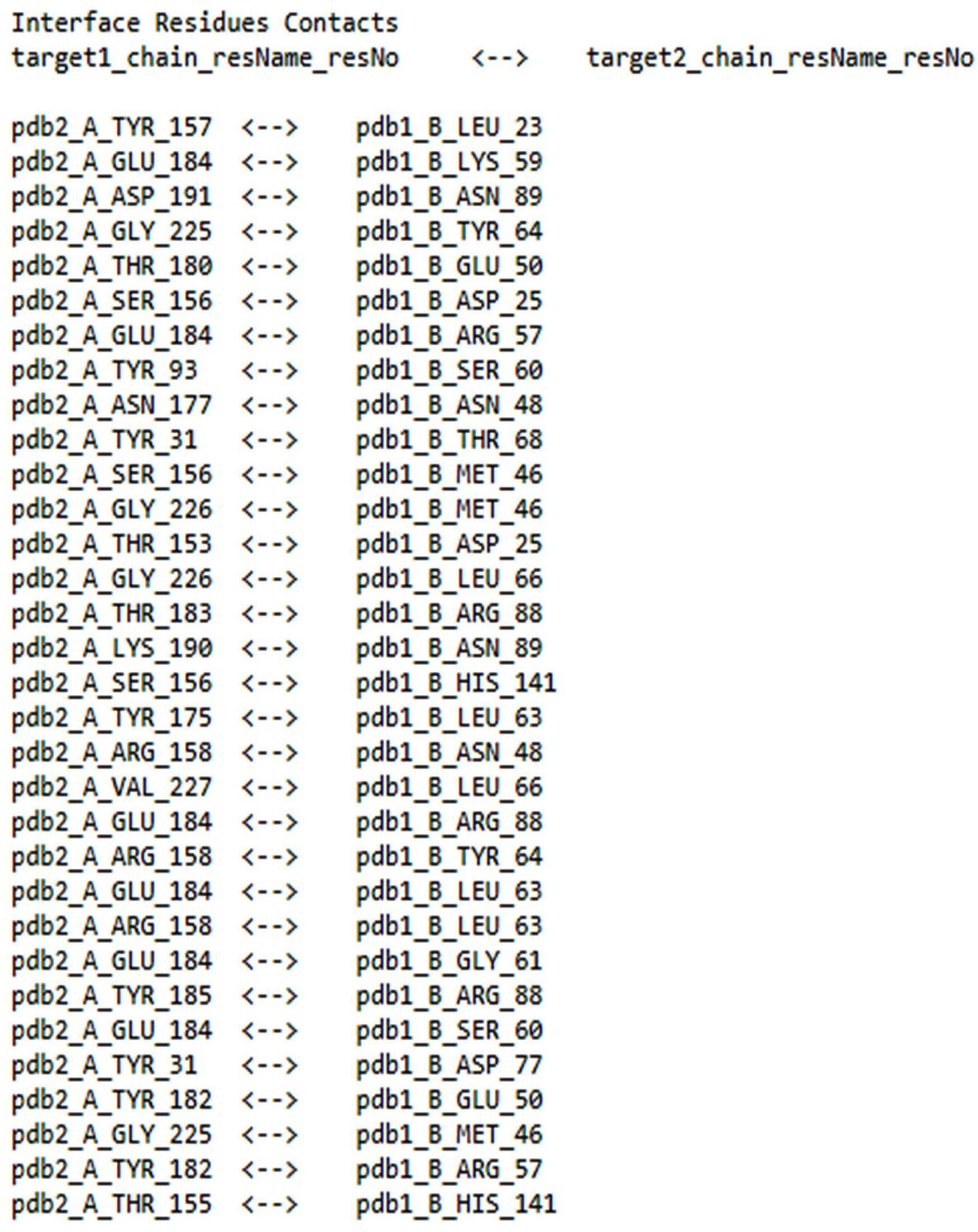
**

**Figure S4.** The fractional accessible surface areas (ASA) were measured for the scFv region by VADAR version 1.8.

**
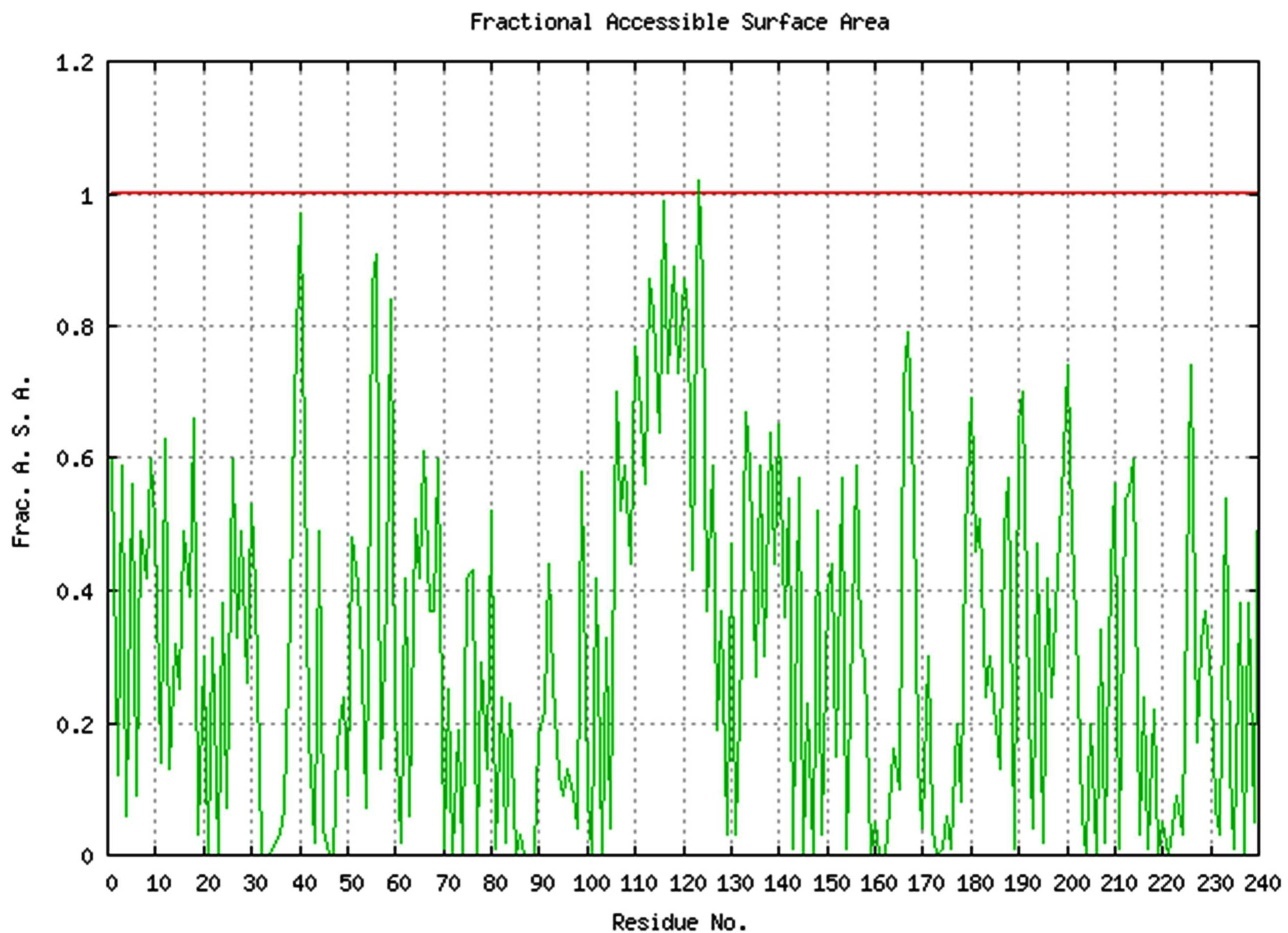
**

**Figure S5.** Using NAPS, distance matrix of the scFv was measured and depicted at the left panel. At the right panel, the scFv structure was illustrated. The distances were colored in Å at the bottom panel.

**
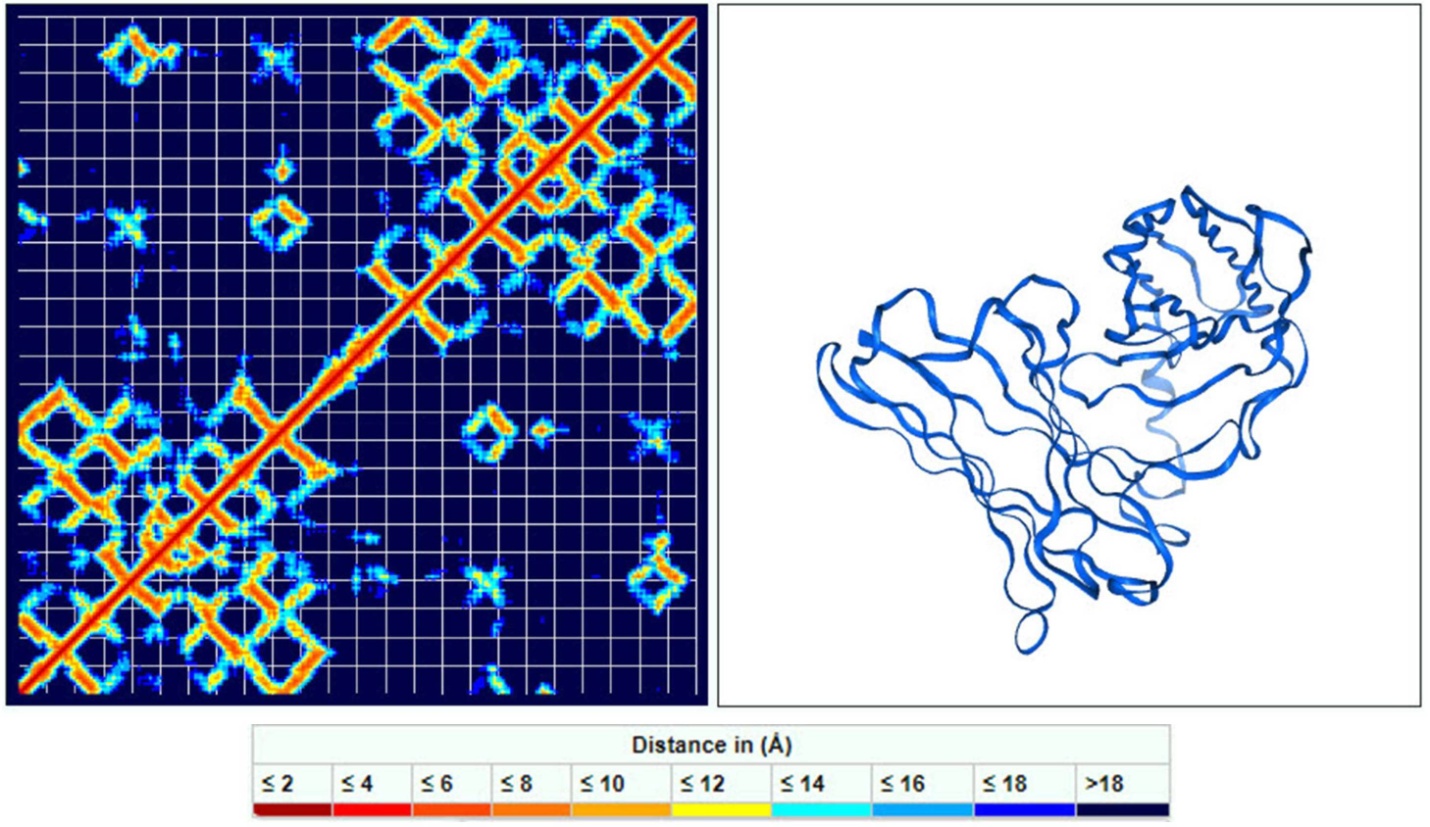
**

**Figure S6.** Contact map of the scFv was illustrated by NAPS (left panel). At the right panel, the scFv structure was illustrated.

**
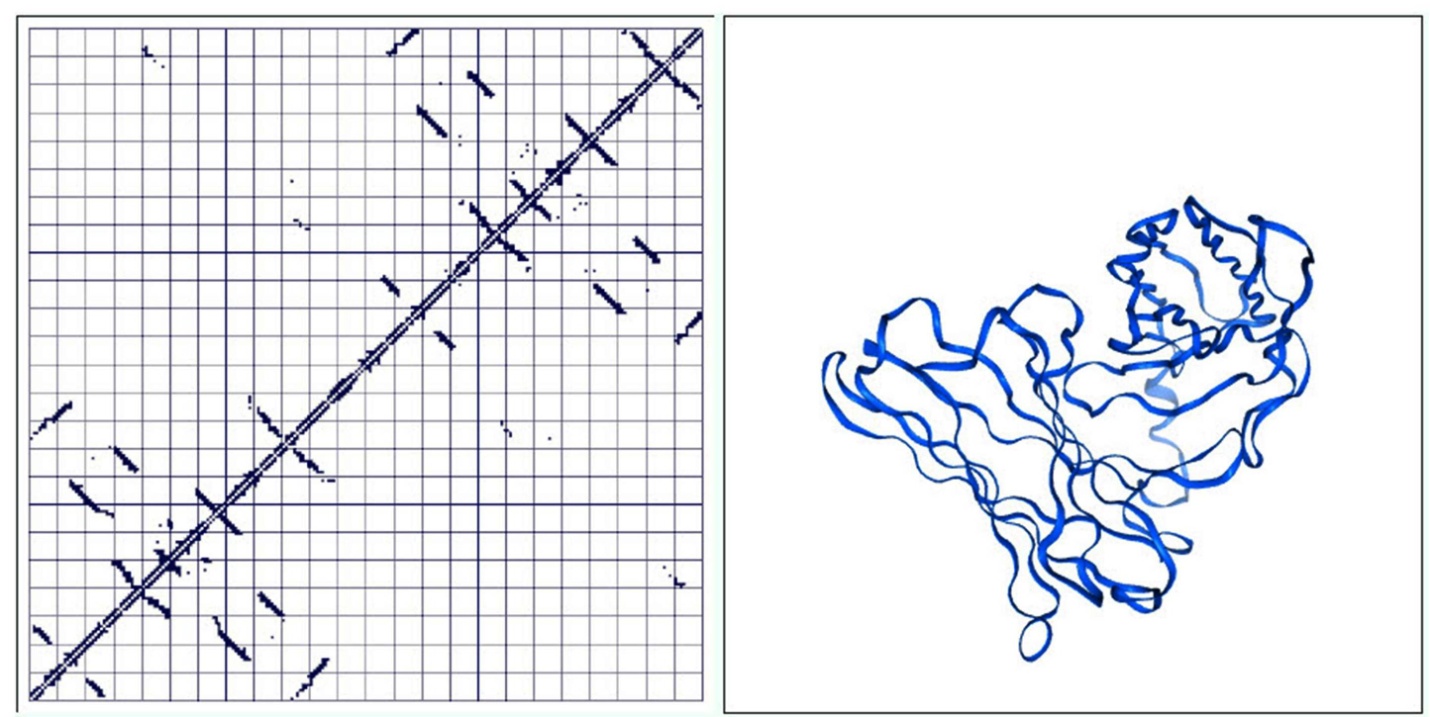
**

**Figure S7.** The number of H bonds during 40000 frames for CDR1 of scFv resulted from Amber V.14 software.

**
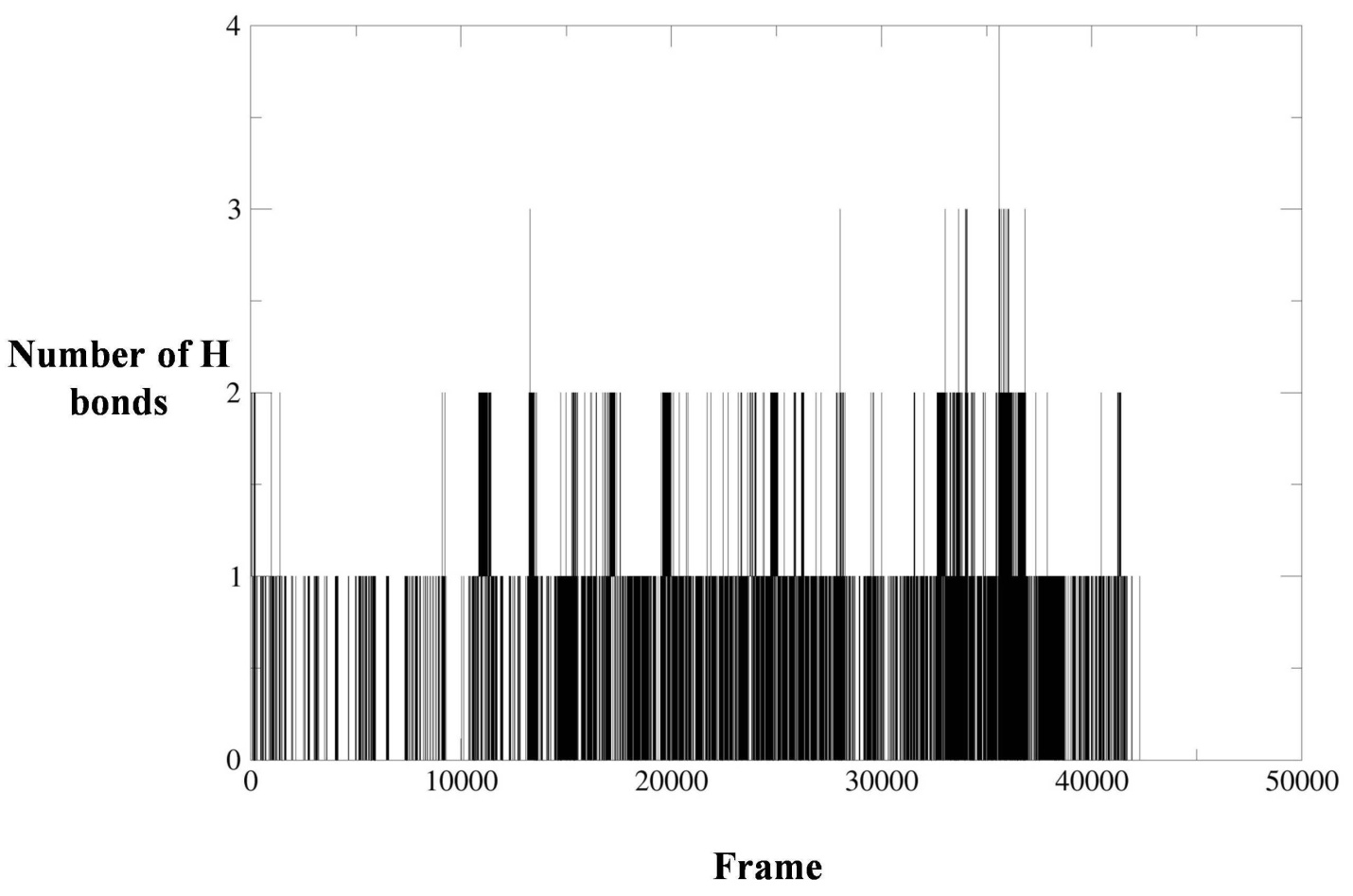
**

**Figure S8.** The number of H bonds during 40000 frames for CDR2 of scFv resulted from Amber V.14 software.

**
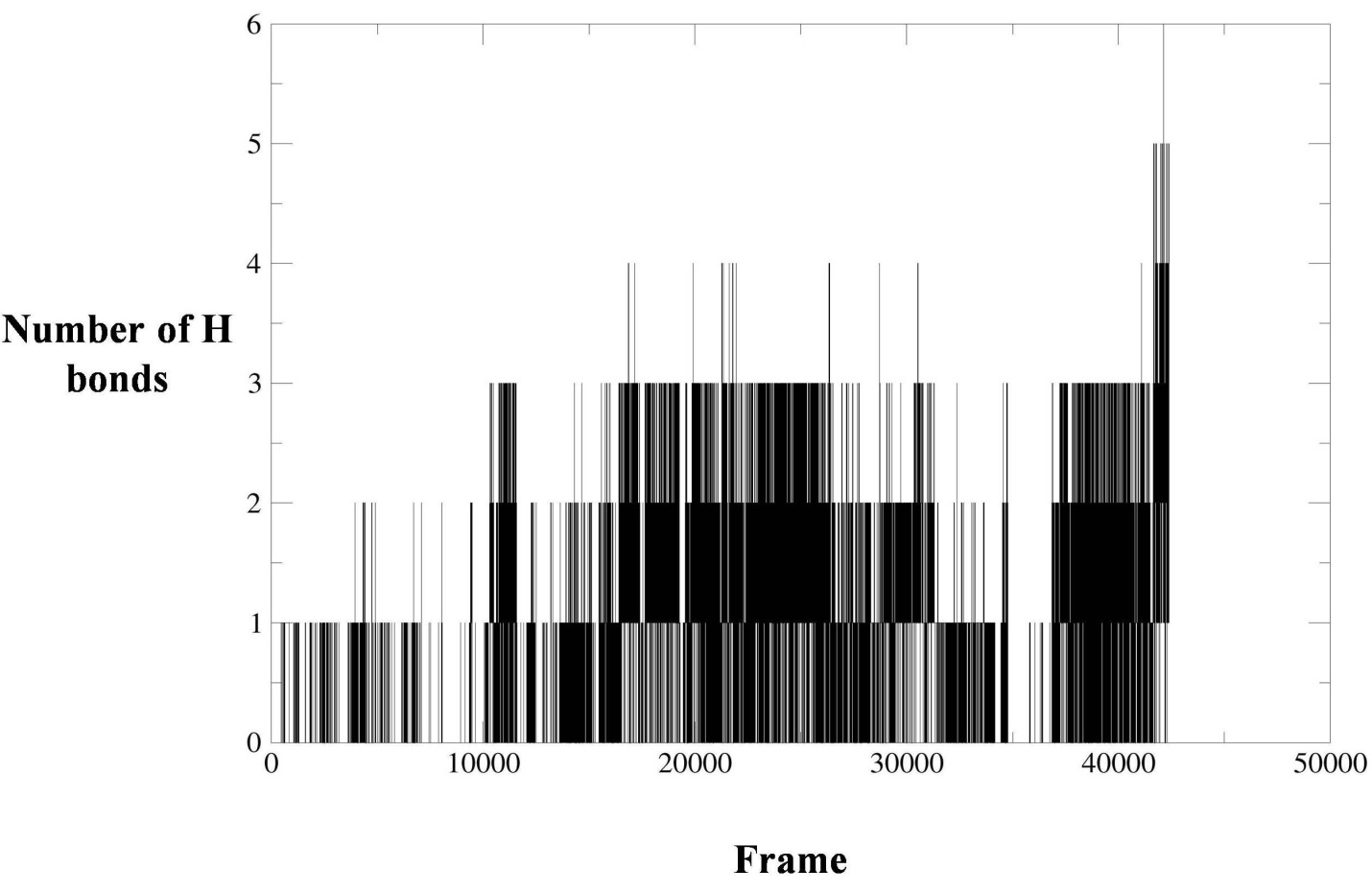
**

**Figure S9.** The number of H bonds during 40000 frames for CDR3 of scFv resulted from Amber V.14 software.

**
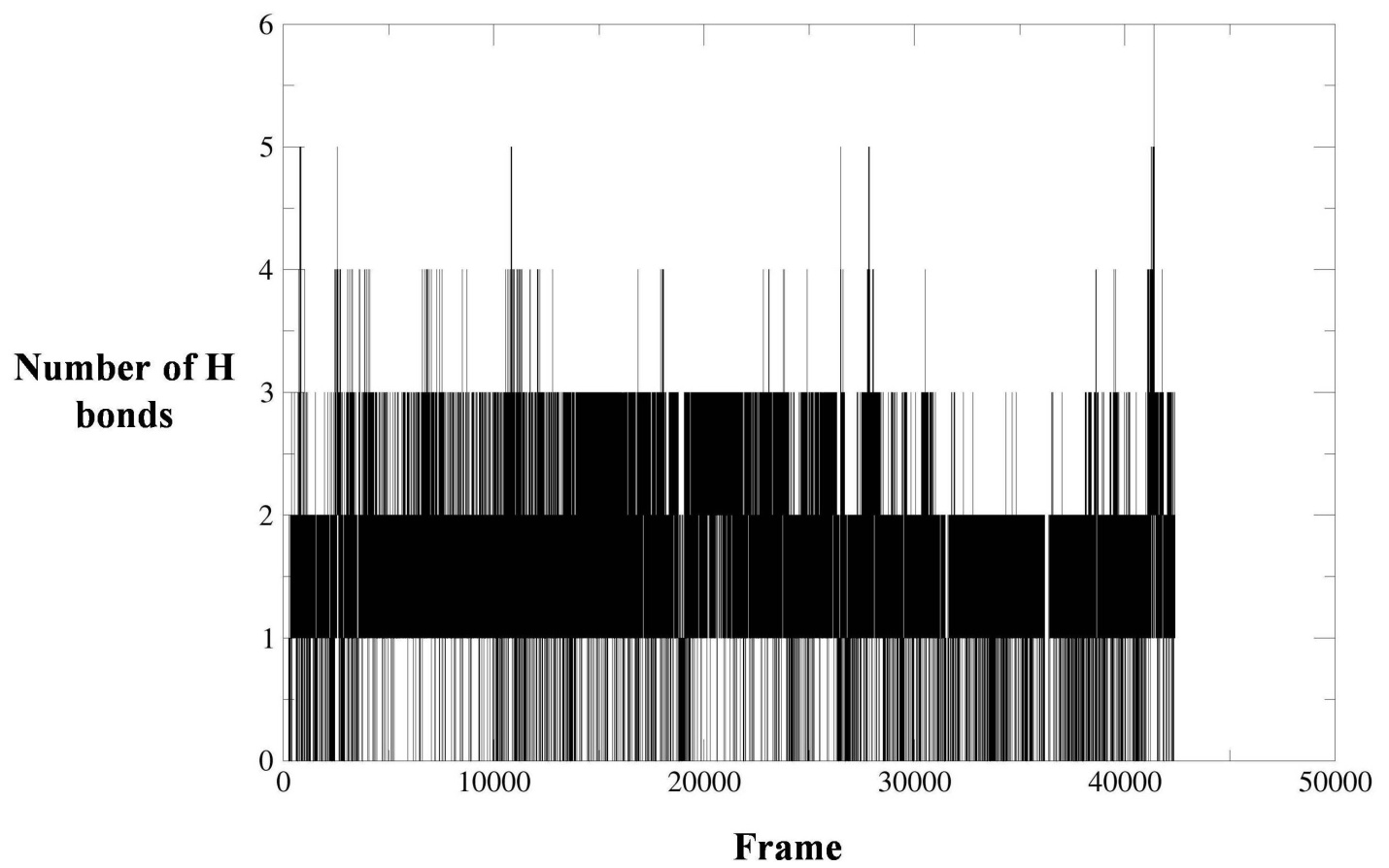
**

**Figure S10.** The number of H bonds during 40000 frames for CDR4 of scFv resulted from Amber V.14 software.

**
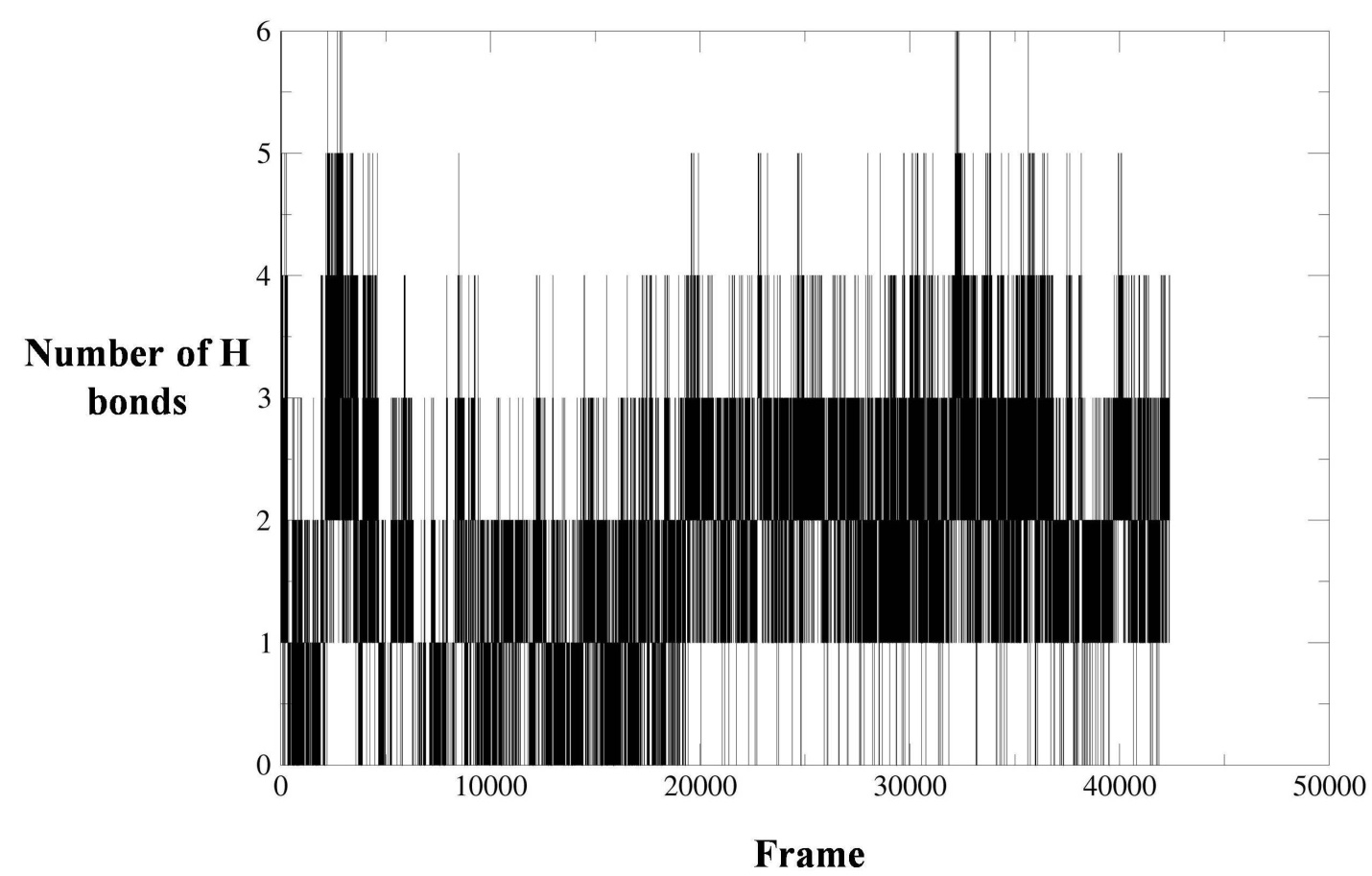
**

**Figure S11.** The number of H bonds during 40000 frames for CDR5 of scFv resulted from Amber V.14 software.

**
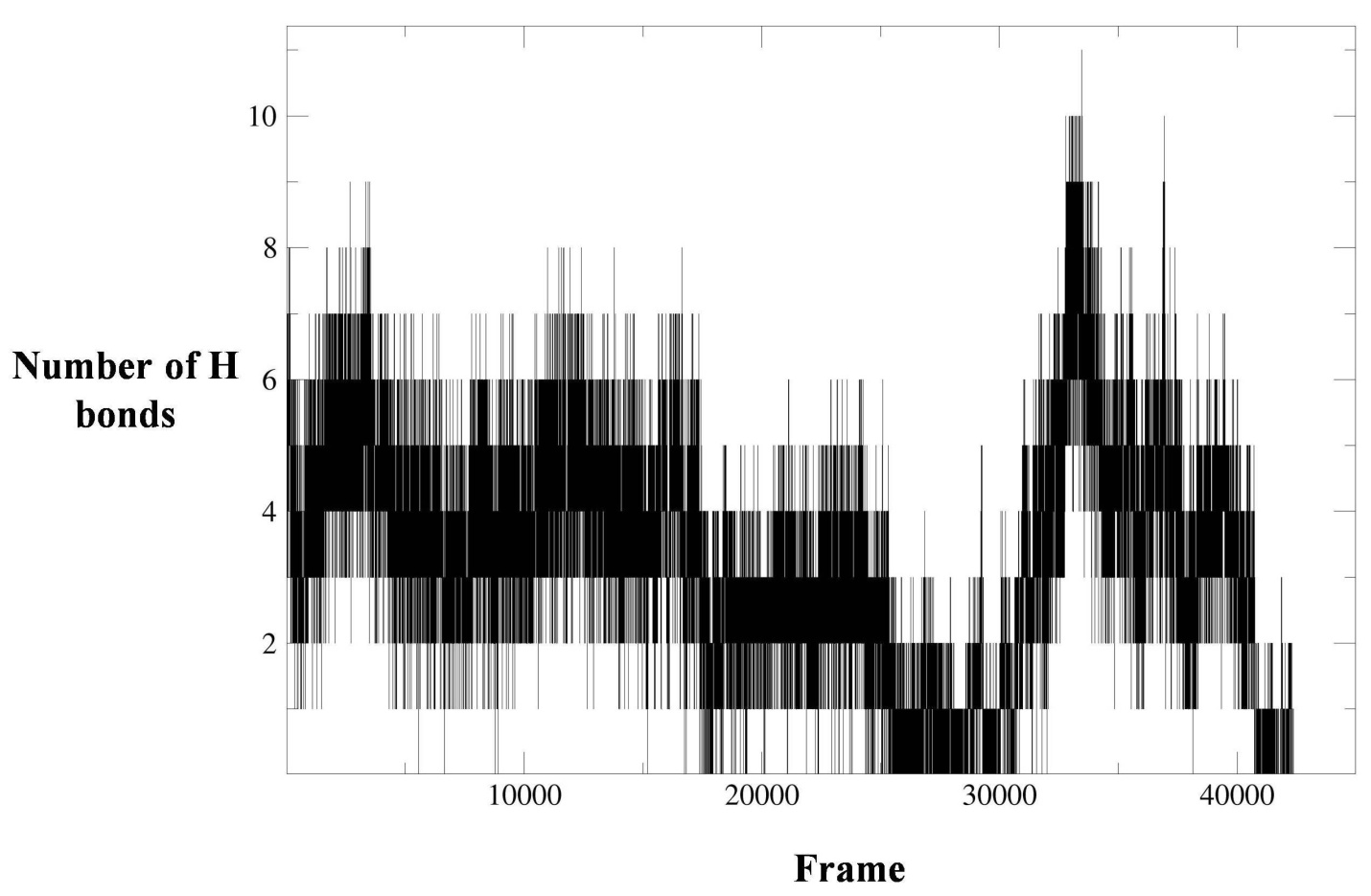
**

**Figure S12.** The number of H bonds during 40000 frames for CDR6 of scFv resulted from Amber V.14 software.

**
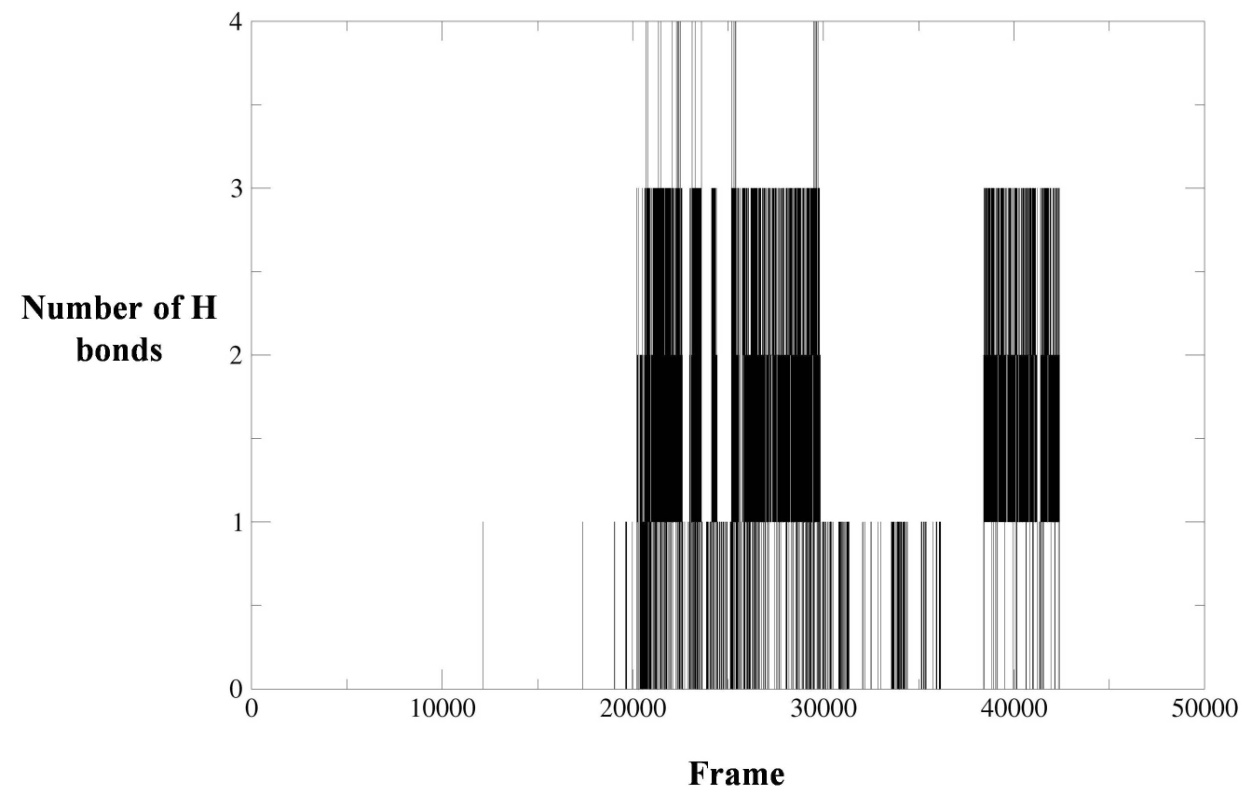
**

**Figure S13.** The calculated molecular weight of anti-CD25 CAR protein by Protein Molecular Weight web server.

**
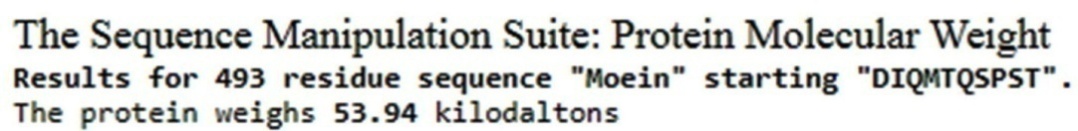
**

**Figure S14.** The prediction of N-linked glycosylation sites in anti-CD25 CAR protein by NetNGlyc 1.0 Server.

**
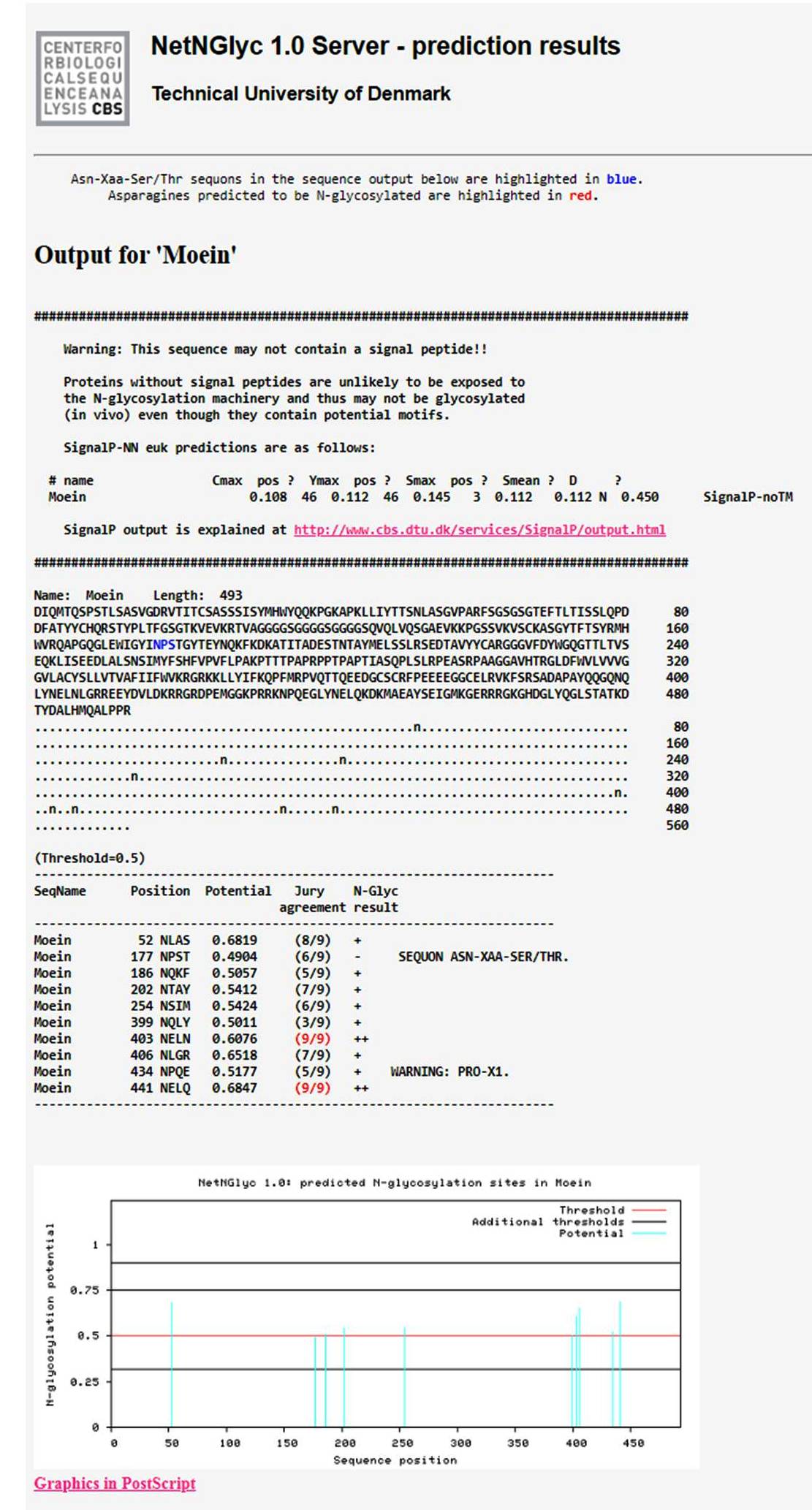
**

**Figure S15.** The prediction of O-linked glycosylation sites in anti-CD25 CAR protein by NetOGlyc 4.0 Server.

**
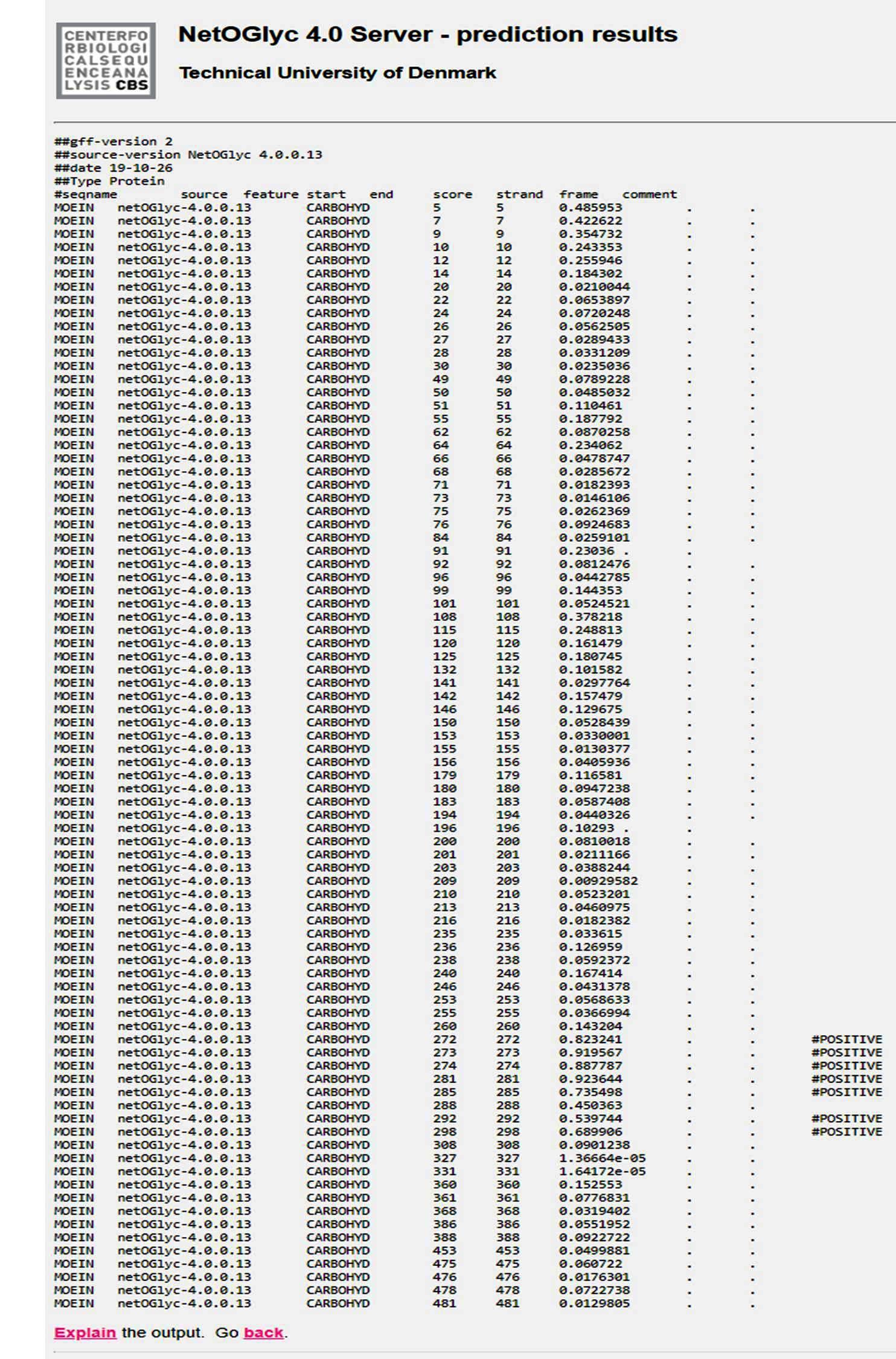
**

**Figure S16.** Flow cytometry analysis of CD25 expression on peripheral blood NK cells. Unstained specimen (A).

**
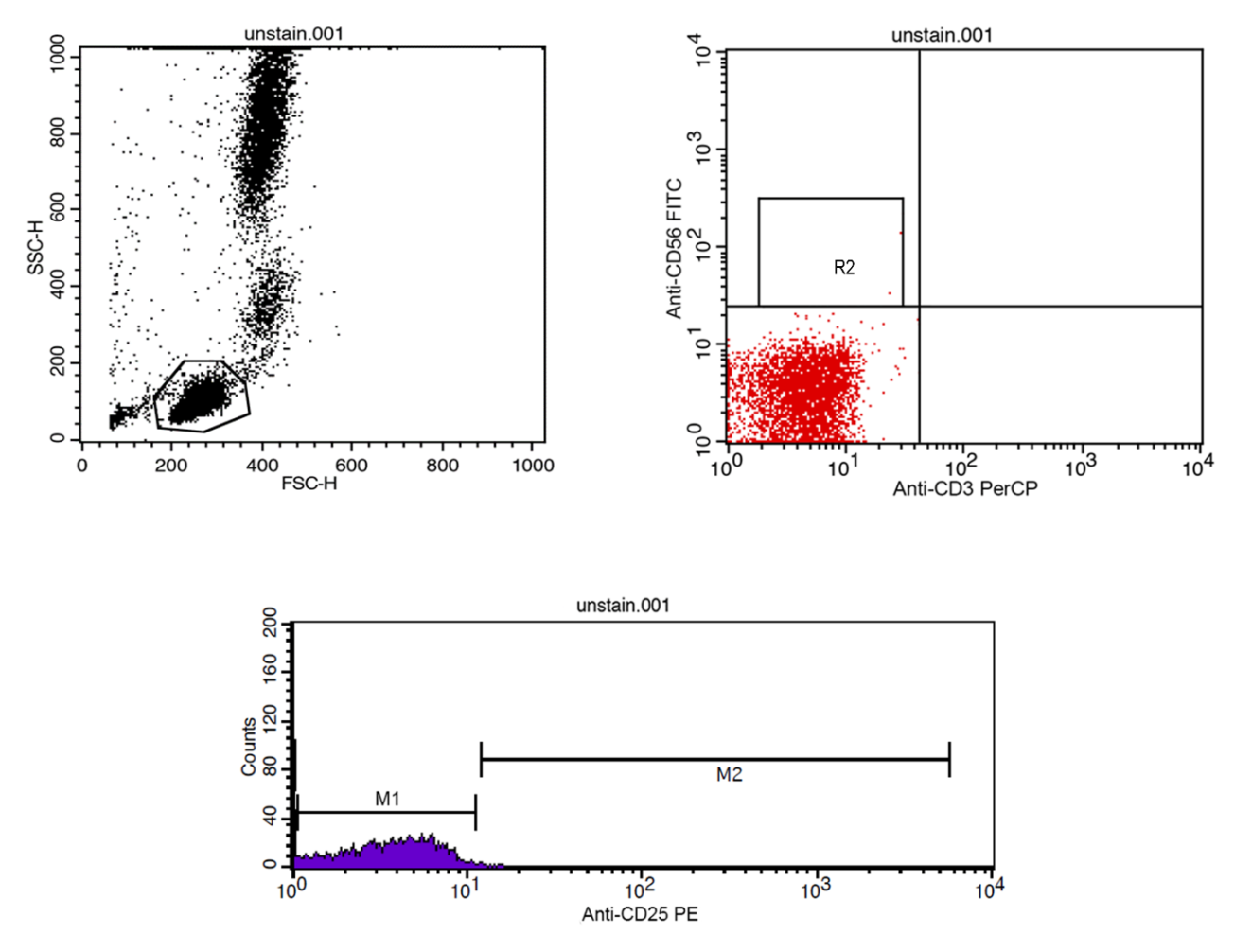
**

Stained with anti CD3-PerCP, anti CD56-FITC and anti CD25-PE (B).


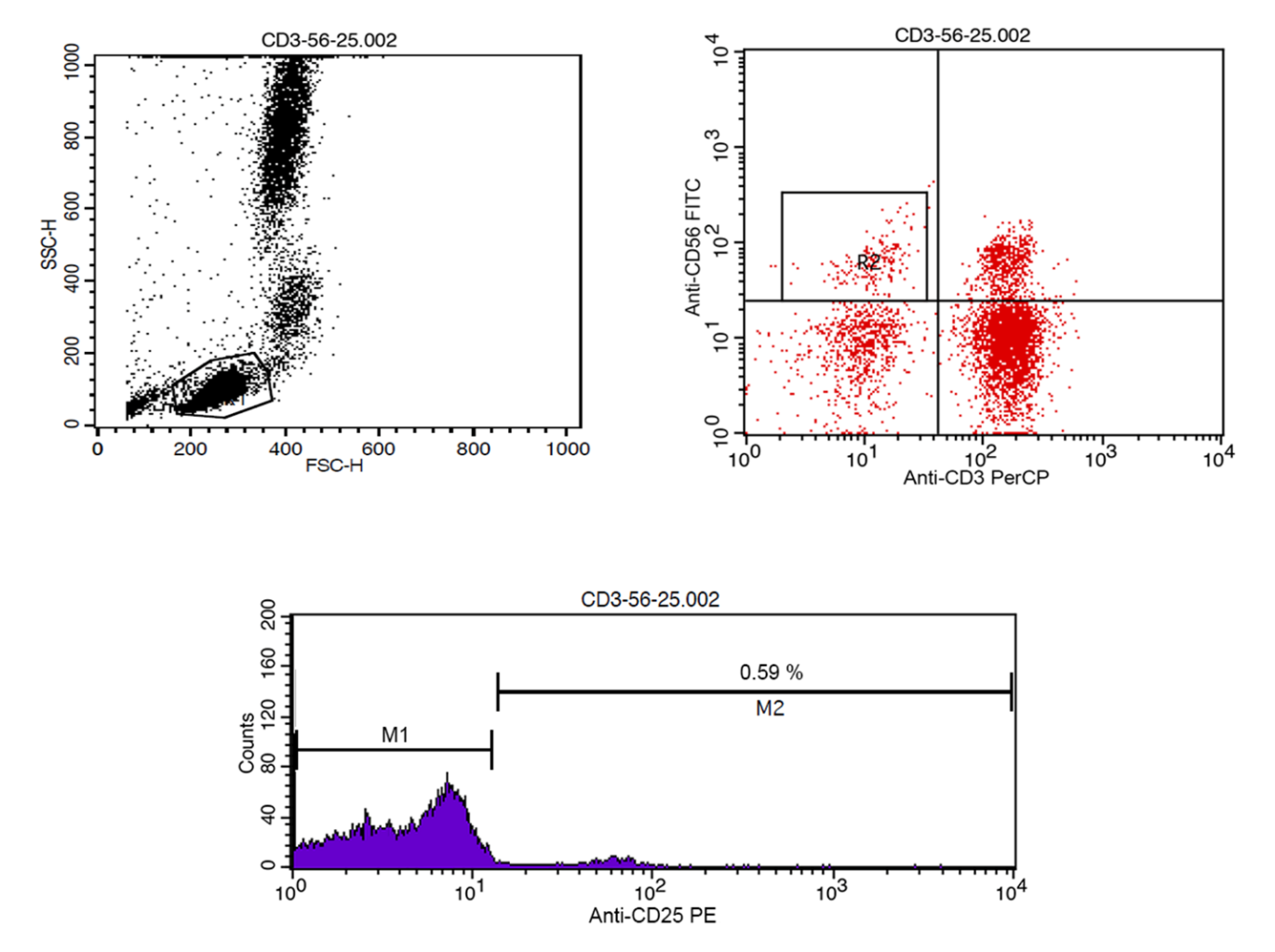


**Table S1.** The resulted torsional angles of scFv (Gly_4_Ser)3 linker.

|  | **Amino acids of linker** | **ϕ** | **ψ** | **ω** |
| --- | --- | --- | --- | --- |
| **1** | GLY111 | -61.21 | -19.37 | 179.98 |
| **2** | GLY112 | -64.55 | -16.83 | 179.18 |
| **3** | GLY113 | -65.43 | 16.98 | 179.44 |
| **4** | GLY114 | -89.95 | 0.21 | -178.30 |
| **5** | SER115 | -63.49 | 137.98 | 177.22 |
| **6** | GLY116 | -93.57 | 5.32 | -177.13 |
| **7** | GLY117 | -96.46 | -164.11 | -179.34 |
| **8** | GLY118 | -112.56 | -161.72 | -179.46 |
| **9** | GLY119 | -98.32 | -171.09 | -179.20 |
| **10** | SER120 | -69.70 | -16.74 | -179.59 |
| **11** | GLY121 | -90.36 | 6.64 | -177.48 |
| **12** | GLY122 | -89.38 | -153.10 | -175.38 |
| **13** | GLY123 | -58.88 | 143.92 | -179.26 |
| **14** | GLY124 | 96.26 | -14.80 | -179.67 |
| **15** | SER125 | -80.33 | 70.18 | -173.30 |
